# Single-nucleotide polymorphisms in genes associated with the vitamin D pathway related to clinical and therapeutic outcomes of American tegumentary leishmaniasis

**DOI:** 10.3389/fcimb.2024.1487255

**Published:** 2025-01-08

**Authors:** Iara Barreto Neves Oliveira, Ramon Vieira Nunes, Vanessa Rafaela Milhomem Cruz Leite, Camila Freire Araújo, Murilo Barros Silveira, Sebastião Alves Pinto, Lorena Andrade Lamounier, Clayton Luiz Borges, Edésio Martins, Iane de Oliveira Pires Porto, Rodrigo Saar Gomes, Fátima Ribeiro-Dias

**Affiliations:** ^1^ Laboratório de Imunidade Natural (LIN), Instituto de Patologia Tropical e Saúde Pública, Universidade Federal de Goiás, Goiânia, Goiás, Brazil; ^2^ Laboratório de Biologia Molecular (LBM), Instituto de Ciências Biológicas, Universidade Federal de Goiás, Goiânia, Goiás, Brazil; ^3^ Department of Infectious Diseases, Hospital de Doenças Tropicais Dr. Anuar Auad, Goiânia, Goiás, Brazil; ^4^ Department of Anatomopathology, Instituto Goiano de Oncologia e Hematologia (INGOH), Goiânia, Goiás, Brazil; ^5^ Department of Dermatology, Clínica São Braz, Goiânia, Brazil; ^6^ Department of Morphofunctional Axis, Universidade de Rio Verde, Goiânia, Goiás, Brazil

**Keywords:** single nucleotide polymorphism, vitamin D receptor, interleukin-32, CYP27B1, interleukin-15, *Leishmania (Viannia)*

## Abstract

**Background:**

The vitamin D pathway contributes to the microbicidal activity of macrophages against *Leishmania* infection. In addition to induction of this pathway, interferon-gamma (IFNγ), interleukin (IL)-15, and IL32γ are part of a network of pro-inflammatory cytokines. The aim of this study was to evaluate single-nucleotide polymorphisms (SNPs) in the components of the vitamin D pathway and associated cytokine genes that could be related to resistance or susceptibility to American tegumentary leishmaniasis (ATL).

**Methods:**

The expressions of *IFNG*, *IL15*, *IL32*, *CYP27B1*, *VDR*, and other pro-inflammatory cytokines *TNF*, *IL6*, and *IL17* genes were evaluated using real-time polymerase chain reaction (qPCR) in lesions of patients with localized cutaneous leishmaniasis (LCL) or mucosal leishmaniasis (ML). SNP genotypes/alleles (in *IL15*, *IL32*, *CYP27B1*, and *VDR*) were evaluated by TaqMan PCR assays using DNA from the blood of patients and healthy individuals. Serum vitamin D levels were determined by chemiluminescence.

**Results:**

Vitamin D pathway-associated genes were expressed in cutaneous as well as mucosal lesions. *IFNG*, *IL6*, and *IL17* were more highly expressed in ML than in LCL. In contrast, *IL32γ/CYP27B1/VDR* mRNAs were mainly correlated in LCL, and *IL32γ* in ML makes strong connections with all cytokines. The SNP *IL32* rs1555001 was less frequent in patients with ML. In addition, some SNPs appear to influence the *VDR* and *CYP27B1* (*IL15* rs10519613 and *IL15* rs3775597) and *IL6* (*VDR* rs7975232) expressions in LCL and the *IL17* expression in ML (*IL15* rs3775597). Gene expression was also correlated with clinical parameters, such as number of lesions (*CYP27B1* mRNA) and treatment failure (*VDR* mRNA). In addition, one SNP was associated with treatment failure in ML (*VDR* rs7975232).

**Conclusions:**

Our findings suggested that some SNPs in the vitamin D pathway-associated genes can be related to resistance and therapeutic outcomes of ATL. They are promising candidates that need to be further evaluated to understand their biological effects in the control or immunopathogenesis of ATL.

## 1 Introduction

American tegumentary leishmaniasis (ATL) is an infectious parasitic disease caused by protozoa of the *Leishmania* genus. In the Americas, Brazil was the country with the highest number of reported cases (12,878) of ATL in 2022 (WHO, 2023). Patients with ATL can present a limited number of cutaneous lesions (one to 10), characterized into localized cutaneous leishmaniasis (LCL) or mucosal lesions, known as mucosal leishmaniasis (ML), in addition to other clinical forms that are less frequent (Goto and Lindoso, 2012). Host immune responses are decisive for the clinical outcome and can help in the successful treatment. These responses begin with the recognition of the protozoan by innate receptors that activate innate immune cells to produce cytokines, which modulate the acquired immune response that is responsible for the control of infection (Gollob et al., 2014). Among pro-inflammatory cytokines, tumor necrosis factor (TNF), interleukin (IL)-6, and IL-1β are produced early during infection. In addition, the pro-inflammatory cytokine IL-32 (Kim et al., 2005) is expressed in lesions of ATL patients (Galdino et al., 2014), and in mice infected with *Leishmania* (*Viannia*) *braziliensis*, the presence of IL32γ isoform contributed to the control of skin lesions (Gomes et al., 2017). Interferon-gamma (IFNγ), a crucial cytokine for macrophage activation, is produced by cells of innate and acquired immunity such as NK cells and type 1 helper T lymphocytes (Th1), respectively. This cytokine increases nitric oxide (NO) and reactive oxygen species (ROS) production to enable macrophages to kill the parasites (Liew et al., 1990; Novais et al., 2014; Scott and Novais, 2016; Toepp and Petersen, 2020). Moreover, IFNγ also induces IL-32 production by human macrophages (Montoya et al., 2014). Another pro-inflammatory cytokine IL-17 appears to play a role in the control (Novoa et al., 2011) as well as the immunopathogenesis of ATL or the progression of infection (Bacellar et al., 2009; Souza et al., 2013).

Vitamin D has been described as a regulator of immune responses during infectious diseases (Athanassiou et al., 2023). The vitamin D-dependent microbicidal activity of monocytes/macrophages has been described in *Mycobacterium tuberculosis* (Fabri et al., 2011; Montoya et al., 2014), *Paracoccidioides brasiliensis* (Guimarães de Matos et al., 2021), and *Leishmania* infection (Crauwels et al., 2019; Silva et al., 2020). In the vitamin D activation pathway, IFNγ induces STAT-1-dependent production of IL-15 in macrophages. This cytokine leads to upregulation of the CYP27B1 enzyme, which catalyzes the conversion of the inactive form of vitamin D [25(OH)D] into the active form [1,25(OH)_2_D_3_]. Additionally, IL-15 increases IL-32 production, which, in turn, induces CYP27B1 (Montoya et al., 2014). The active form of vitamin D activates the vitamin D receptor (VDR), which binds to VDR elements (VDREs) in the target genes, leading, e.g., to the production of antimicrobial peptides (AMPs). These AMPs, cathelicidin and β-defensin-2, act directly to control microorganisms. Crauwels et al. (2019) showed that vitamin D activates the transcription of the cathelicidin gene (*CAMP*) *in vitro* in human macrophages infected with *Leishmania* spp., contributing to infection control. Our group has demonstrated that IL-15 and IL-32 work together to increase the leishmanicidal activity of human macrophages through the production of ROS in a vitamin D-dependent manner (Silva et al., 2020). Furthermore, in ATL lesions of *L.* (*V.*) *braziliensis*-infected patients, transcriptional data demonstrated increase of cytokines and vitamin D pathway gene expression such as *TNF* (Christensen et al., 2016), *IL1B* (Amorim et al., 2019), *IL32* (Galdino et al., 2014; Novais et al., 2015), *IL15*, *IFNG*, *VDR*, *CYP27B1* (Novais et al., 2015), and *IL17* (Katara et al., 2013).

The immune response can be affected by host genetic variability such as single-nucleotide polymorphisms (SNPs), which consist of exchanging one nucleotide for another, altering or not the amino acid sequence. Depending on the change in the immune genes, the expression and/or function of the protein can be affected, altering the immune responses and, consequently, the outcome of the diseases (Cabrera et al., 1995; Salhi et al., 2008). Concerning New World *Leishmania* studies, it has been demonstrated that SNPs in the *CXCR1* and *CXCR2* (Castellucci et al., 2010), *FLI1* (Castellucci et al., 2011), *TGFBR2*, *SMAD2*, *SMAD3*, *SMAD7* (Castellucci et al., 2012), *COL1A1* (Almeida et al., 2015), *IL1B* (da Silva et al., 2019), and *IFNG-AS1* (Castellucci et al., 2021) genes are associated with risk to ATL.

Shabandoust et al. (2020) evaluated polymorphisms (*Bsm*I, *Taq*I, and *Fok*I) in the *VDR* gene in patients with LCL infected with *Leishmania tropica* (Old World *Leishmania*), but no association with susceptibility or resistance to the disease was found. In our previous study, two SNPs in the *IL32* gene were associated with protection or susceptibility to ATL (Dos Santos et al., 2020). Recently, Salem et al. (2023) identified, in Saudi patients, the association of polymorphisms of the *VDR* gene with parasite load and susceptibility to cutaneous leishmaniasis caused by Old World *Leishmania*.

This study first identified the expression of vitamin D pathway-associated genes as well as pro-inflammatory genes in patients with ATL. Further, as SNPs in genes of the components of the vitamin D pathway can influence the outcome of *Leishmania* infection, the present study aimed to evaluate some SNPs in genes of the vitamin D pathway (*IL15*, *IL32*, *CYP27B1*, and *VDR*), in association with clinical and treatment outcomes. The study can identify genetic markers of susceptibility or resistance to ATL as well as new candidate genes to be studied in the immune response against *Leishmania*.

## 2 Materials and methods

### 2.1 Study design, samples, and ethical aspects

**Figure 1:**
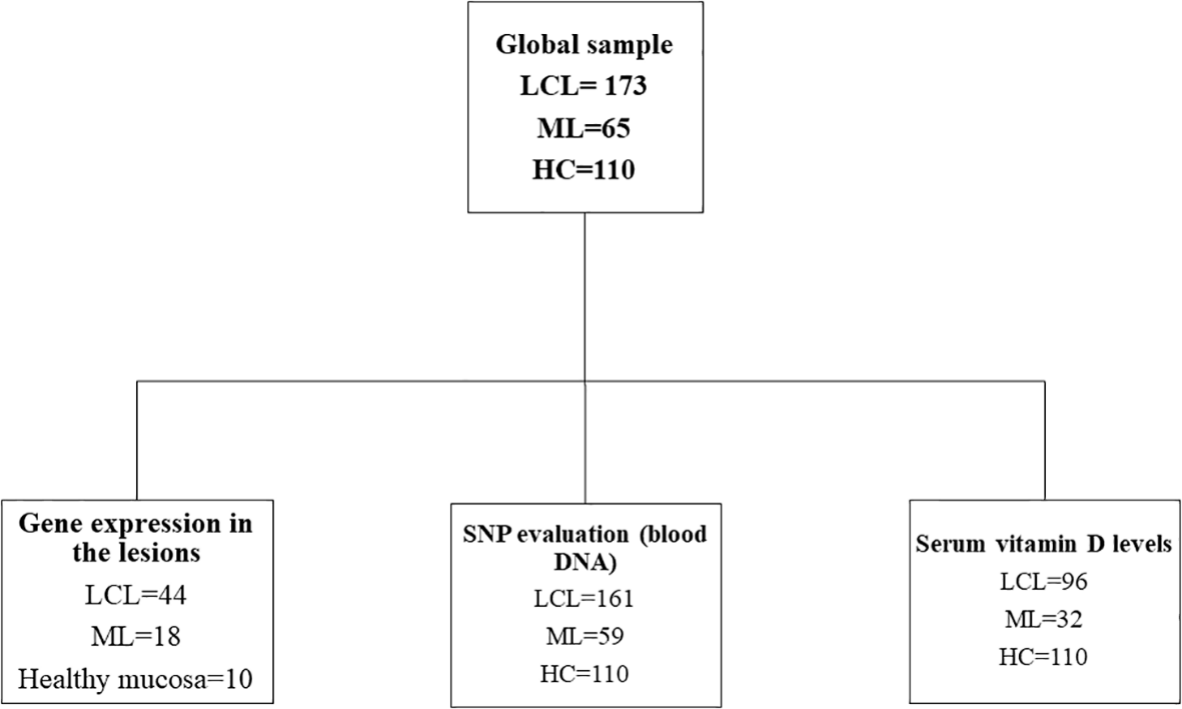
Study design of patients with American tegumentary leishmaniasis (ATL; n = 238) and healthy controls (HC; n = 110). Samples were obtained from 2017 until 2022 in Hospital Annuar Auad, Goiânia, Goiás. Samples of patients with localized cutaneous (LCL) or mucosal leishmaniasis (ML) were analyzed for gene expression (by real-time PCR; fragment of lesion), serum vitamin D levels, and single-nucleotide polymorphism (SNP; blood DNA).

A flowchart of the study design is shown in **Figure 1**. For this study, a total of 238 patients with ATL (LCL = 173; ML = 65) were assisted and followed up from February 2017 until September 2022 in Annuar Auad Tropical Disease Hospital (HDT/HAA), in Goiânia, Goiás, Brazilian Midwestern region. Patients with ATL were recruited during their attendance in the hospital outpatient clinic, and the healthy control (HC; n = 110) group was composed of individuals from the urban community of Goiânia city, a non-endemic area of leishmaniasis. The controls presented no previous history of leishmaniasis and were matched by their sex and age with those of the patients. From this group, peripheral blood was collected for genetic study (n = 110) and determination of vitamin D serum levels (n = 110).

A case of leishmaniasis was defined by clinical and epidemiological data compatible with the disease, with at least one positive parasitological exam as the direct exam, polymerase chain reaction (PCR) to detect *Leishmania* DNA, or histopathological exam with immunohistochemistry (IHC) for amastigote forms (according to the guidance of BRASIL, 2017). In addition, indirect immunofluorescence (IFI) and Montenegro skin test (MST) were also used for diagnosis according to Pereira et al. (2009). The inclusion criteria for patients were as follows: confirmed diagnosis of ATL, active lesion or not, specific treatment or not, age older than 18 years, male or female sex, and no comorbidities nor use of any anti-inflammatory or antibiotic drugs for 3 weeks before the recruitment. Fragments of the lesions were tested for parasite DNA using PCR–restriction fragment length polymorphism (PCR–RFLP) described by De Andrade et al. (2006), and all results were compatible with *Leishmania Viannia* subgenus. All procedures were approved by the Ethics Committee of HDT/HAA (CAAE n. 81316417.1.3001.0034) and the Hospital of Clinics/UFG (CAAE n. 81316417.1.0000.5078 and 59615915.4.0000.5078), Goiânia, Goiás, Brazil. Informed consent was signed by all patients and controls.

To access gene expression in 62 ATL patients, one fragment of cutaneous (LCL, n = 44) and mucosal (ML, n = 18) lesion edges from patients without any treatment were obtained (from 2017 to 2022) using 5.0-mm punch at the time of ATL diagnosis and stored in TRIzol reagent (Invitrogen, Carlsbad, CA, USA) in the *Leishbank* [Laboratory of Natural Immunity at Institute of Tropical Pathology and Public Health of Federal University of Goiás (IPTSP/UFG)], Goiânia, Goiás. The characteristics of these groups of patients are shown in **Supplementary Table S1**.

To access genetic variations, ATL patients (n = 220) were asked to provide a peripheral blood sample (4 mL) at the time of diagnosis, in addition to two fragments of the lesions. These patients were diagnosed with LCL (n = 161) or ML (n = 59) and were assisted between 2019 and 2022 in HDT/HAA (**Supplementary Table S2**). These patients were still treatment-naïve or had not undergone a previous treatment regimen.

For the evaluation of vitamin D serum levels (by chemiluminescence reaction), peripheral blood samples (n = 110) from 96 LCL and 32 ML patients (from 2019 to 2022) were obtained (**Supplementary Table S2**). These patients were only treatment-naïve. Serum vitamin D levels and gene expression levels were evaluated in the lesions of 32 patients with LCL and 12 with ML. Evaluation of gene expression levels in the lesions and SNPs was performed in 28 patients with LCL and nine with ML. All patients with LCL (n = 96) and LM (n = 32) with vitamin D measurements also underwent SNP genotyping. Among the three groups, there were 32 patients with LCL and 12 with LM in common.

### 2.2 Clinical characteristics of patients with cutaneous or mucosal lesions

The clinical parameters of patients, included in the transcriptional and genetic polymorphism evaluation, were as follows: number and total area (cm^2^) of lesions (for LCL), type of lesions, and duration of disease (for all patients). The cutaneous lesions were identified as ulcerated, crusted ulcer, nodular, vegetative ulcer, and/or infiltrative papules, whereas the mucosal lesions were characterized as ulcerated, erythematous, infiltrative, and/or nasal septum perforation. For each lesion, the size was measured longitudinally and vertically to determine the area in cm^2^ (**Supplementary Tables S1–S3**).

### 2.3 Transcriptional analysis

#### 2.3.1 Public transcriptome data analyses

Previously published microarray data of normal skin samples (n = 10) and LCL (n = 17) lesion samples were obtained from the publicly available National Center for Biotechnology Information (NCBI) Gene Expression Omnibus (GEO) database (accession number GSE55664) (Novais et al., 2015). The expression levels of selected genes in normal skin versus *L.* (*V.*) *braziliensis*-caused lesions were represented in a heatmap as well as median, interquartile, maximum, and minimum values. The correlation analysis of the expression levels of genes was performed, and the data were presented by heatmaps.

#### 2.3.2 Gene expression analyses by real-time polymerase chain reaction

RNA was extracted from lesions of patients with ATL using the TRIzol method (Invitrogen, Waltham, MA, USA) with isopropanol following the manufacturer’s instructions. cDNA was made using the iScript kit (Bio-Rad, Hercules, CA, USA). The *TNF*, *IL6*, *IFNG*, *IL15*, *IL32γ*, *IL17*, *CYP27B1*, and *VDR* primer sequences were pre-designed primers KiCqStar Sigma-Aldrich (St. Louis, MO, USA) and are described in **Supplementary Table S3**. Diluted cDNA was used for real-time polymerase chain reaction (qPCR), which was performed using the QuantStudio Real-Time PCR system (Thermo Fisher Scientific, Waltham, MA, USA) with SYBR Green Mastermix (Applied Biosystems, Foster City, CA, USA). Relative expressions were calculated using the 2^−ΔΔCT^ method and normalized against the housekeeping gene GAPDH (Galdino et al., 2014).

### 2.4 Determination of serum levels of vitamin D

From blood collected in a tube without anticoagulant, the serum was separated after centrifugation (600 g, 10 min, 4°C) and used to determine the vitamin D levels. The chemiluminescence technique was performed according to the manufacturer’s instructions (Beckman Coulter, Brea, CA, USA) in cobas^®^ 6000 (Roche, Basel, Switzerland). Patients and controls were divided into subgroups according to vitamin D levels, which were vitamin D sufficiency (≥30 ng/mL) or insufficiency (from 20 to 29 ng/mL) together with deficiency (<20 ng/mL), according to International Endocrine Society criteria (Holick, 2009) in agreement with the update on vitamin D deficiency reported by Amrein et al. (2020).

### 2.5 Isolation of genomic DNA and genetic polymorphism assessment

DNA was isolated from peripheral venous blood of patients with ATL and healthy controls using the illustra blood genomicPrep Mini Spin Kit (GE Healthcare, Little Chalfont, UK), according to the manufacturer’s protocol. Three replicates of the sample were stored for the next step. SNPs in the *IL15*, *IL32*, *VDR*, and *CYP27B1* genes were selected based on the frequency in the Latin American population, previously described in association with human infectious diseases, at the National Center for Biotechnology Information SNP database (http://www.ncbi.nlm.nih.gov/snp/). Genotyping of patients with ATL and controls was performed by TaqMan SNP assays (**Supplementary Table S4**), according to the manufacturer’s protocol, using the QuantStudio Real-Time PCR system (Thermo Fisher Scientific, Waltham, MA, USA). Quality control was performed by the incorporation of positive and negative controls.

### 2.6 Treatment of patients and follow-up for clinical and therapeutic outcomes

Treatment was offered to all patients following the guidelines of the Brazilian Ministry of Health (Brazil, 2007), in which pentavalent antimonial (meglumine antimoniate, 20 mg/kg/day for 20 days) and liposomal amphotericin B are the first-option drugs, followed by fluconazole or itraconazole as the second option in cases of contraindication. Successful therapeutic outcome means the clinical cure of patients whose (re-epithelialization) cutaneous or mucosal lesions completely healed within 3 months after the first treatment schedule. Therapeutic failure refers to patients who healed after 3 months and/or remained under medical follow-up with partial improvement or worsening of the lesions. Time to clinical healing consists of the time required after the end of the treatment schedule until the lesions are fully healed. Patients were discharged with a clinical cure after a follow-up of 12 months or longer as needed.

### 2.7 Statistical analysis

The statistical analyses and graphs of mRNA expression in the lesions and vitamin D levels were conducted using the GraphPad Prism 8.01 software (GraphPad Software, San Diego, CA, USA). Data are shown as median, interquartile, maximum, and minimum values. The non-parametric Wilcoxon and Mann–Whitney tests were used to compare paired and unpaired data, respectively. Spearman’s correlation test was applied for association analysis.

The mRNA relative expression in lesions of patients was evaluated in association with clinical characteristics and therapeutic outcomes. Statistical analyses as well as data processing were carried out through the characterization of continuous and categorical variables, analyzing measures of central tendency and dispersion. Friedman’s test was used for non-parametric distribution to compare linked sample data (when the same individual is evaluated more than once time) not using the numerical data directly, but rather the positions occupied by them after the ordering made for each group separately. Additionally, the F test of Levene’s test was used to assess whether the variances of a variable are equal between two or more groups. Statistical analyses were carried out using the STATA^®^ software, v. 14.

The statistical analyses of SNP distribution were performed using the RStudio software (v. 1.0.153) and considered significant when p < 0.05. Expected frequencies of the gene polymorphisms (*IL15* rs10519613, *IL15* rs3775597, *IL32* rs1555001, *IL32* rs2239303, *IL32* rs4349147, *CYP27B1* rs4646536, *VDR* rs7975232, and *VDR* rs2248098) were estimated using the Hardy–Weinberg equilibrium (HWE), and the comparison of the observed and expected genetic frequencies was calculated by Fisher’s exact test (**Supplementary Table S5**). The frequencies of genotypes/alleles were compared between patients with ATL and healthy controls and between patients with LCL or ML using Genepop. For haplotype inference [for linkage disequilibrium (LD) calculation], an expectation–maximization (EM) calculation was performed using the Harlequin software. The LD plot was performed using Haploview. The effect of the genotypes on ATL susceptibility was estimated by calculating odds ratios (ORs) and their 95% confidence intervals, and this analysis was conducted using the GraphPad Prism 8.01 software (GraphPad Software, San Diego, CA, USA). Overall, statistical test *p*-values <0.05 were considered to be statistically significant.

## 3 Results

### 3.1 Expression of vitamin D pathway components is increased in lesions of patients with cutaneous or mucosal leishmaniasis: association with pro-inflammatory cytokines

**Figure 2:**
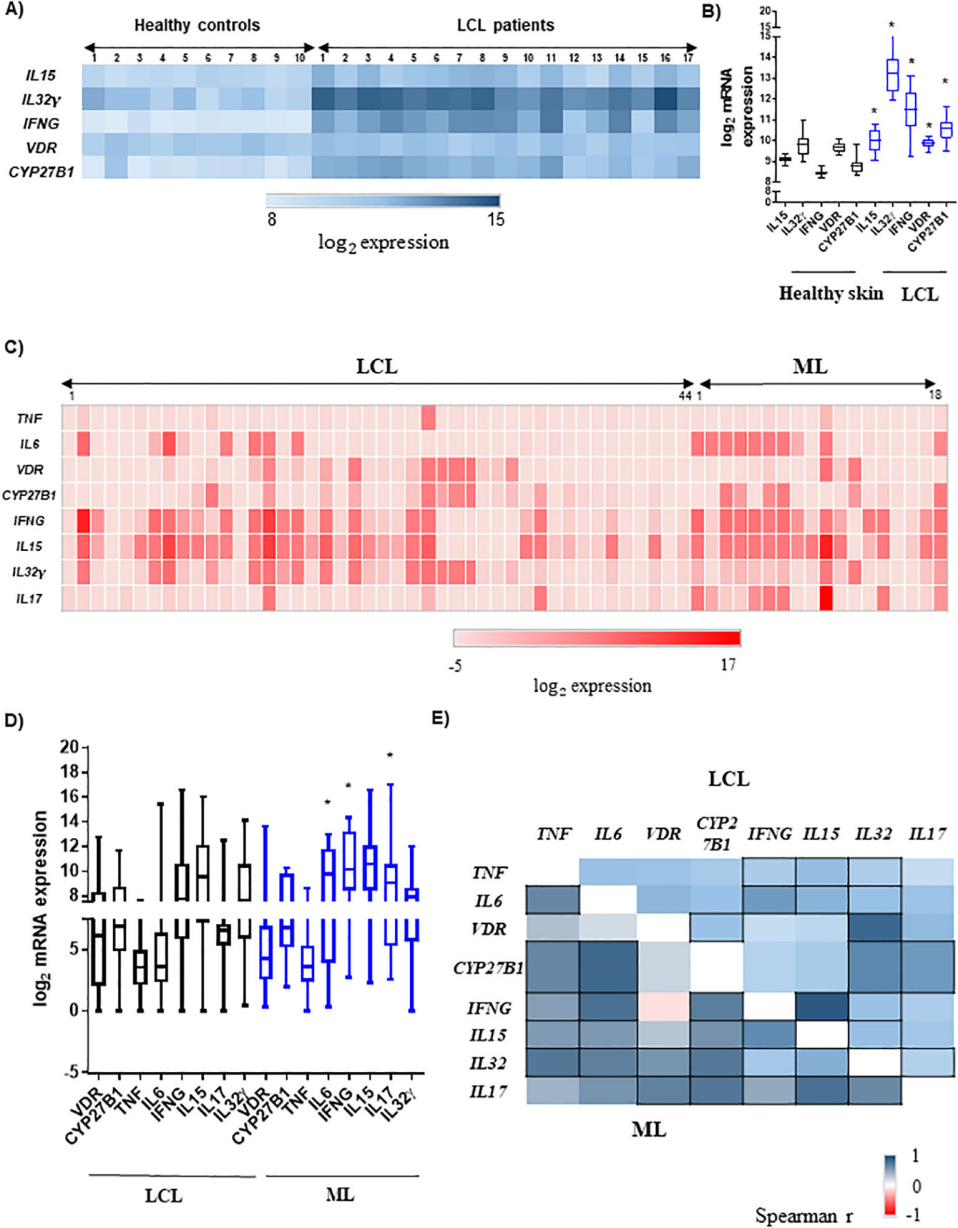
Expression of pro-inflammatory cytokines and the vitamin D pathway components in cutaneous or mucosal leishmaniasis lesions. **(A)** Heatmap of mRNA expression of *IL15*, *IL32*, *IFNG*, *VDR*, and *CYP27B1* in the healthy skin (n = 10) and lesions of localized cutaneous leishmaniasis (LCL; n = 17) patients. **(B)** Gene expression levels in skin from healthy controls (black boxes) and in lesions of LCL patients (blue boxes). The data are presented as median, *p < 0.05 (Mann–Whitney test); transcriptome public data from Novais et al. (2015). **(C)** Heatmap of *TNF*, *IL6*, *VDR*, *CYP27B1*, *IFNG*, *IL15*, *IL32*, and *IL17* mRNA expressions in the lesions of our patients with LCL (n = 44) or mucosal leishmaniasis (ML; n = 18). The scale of relative expression to GAPDH gene is shown as log_2_. **(D)** Gene expression in lesions of our patients with LCL (n = 44) or ML (n = 18). The scale of relative expression to GAPDH gene is shown as log_2_. *p < 0.05 (Mann–Whitney test). **(E)** Heatmap of correlation between the *TNF*, *IL6*, *VDR*, *CYP27B1*, *IFNG*, *IL15*, *IL32γ*, and *IL17* mRNA expression in lesions of our patients. LCL (n = 44) is presented on the top triangle, and ML (n = 18) is presented on the bottom triangle. Spearman’s correlation test was applied, and the scale of Spearman’s r was from −1 until 1. Correlations with p < 0.05 are represented by squares with black borders.

To identify the vitamin D pathway components as possible candidates for resistance against or susceptibility to ATL, we first analyzed previously published transcriptome data of healthy skin and leishmaniasis cutaneous lesions obtained at the moment of disease diagnosis. We detected a statistically significant higher transcriptional level of the vitamin D pathway-associated genes (*VDR*, *CYP27B1*, *IL15*, *IL32γ*, and *IFNG*) in cutaneous lesions compared to healthy skin (**Figures 2A, B**). We confirmed the expression of these genes in lesions of our patients with ATL, and the PCR assays in lesions of all patients identified *L.* (*Viannia*) parasites (data not shown). We found that *IL6*, *IFNG*, and *IL17* mRNA expression levels were higher in patients with ML than in those with LCL (**Figures 2C, D**). For the other genes related to the vitamin D pathway except *IFNG*, the differences were not statistically significant (**Figure 2D**). From this point, we just evaluated patients from our cohort.

We investigated the correlations between the relative mRNA expression of each cytokine and the vitamin D pathway components in the lesions. In LCL patients, there was a positive correlation between *VDR* and *CYP27B1* mRNA levels (r = 0.63; p < 0.001; **Figure 2E**). The expression of these genes was positively associated with *IL32γ* mRNA levels, whereas *CYP27B1* was additionally associated with *IL17* mRNA expression levels (p < 0.05, **Figure 2E**). The mRNA expression levels of *IL32γ*, *IL15*, and *IFNγ* were positively associated with LCL lesions (p < 0.001, **Figure 2E**). In addition, other pro-inflammatory cytokines were significantly associated with these cytokines, such as *TNF* and *IL6*. It is known that the cytokines IL-17 and IL-32 induce each other (Moon et al., 2012), and in LCL lesions, we could detect that *IL17* mRNA levels presented positive correlations with *CYP27B1*, *IFNG*, *IL15*, *IL32γ*, and *IL6* (p < 0.05; **Figure 2E**).

In contrast to LCL lesions, the expression levels of *VDR* and *CYP27B1* mRNA were not significantly correlated in lesions of patients with ML. However, the expression levels of both genes were positively associated with *IL32* (vs. *VDR* r = 0.60 and vs. *CYP27B1* r = 0.76; p < 0.05) as well as with *IL17* mRNA levels (vs. *VDR* r = 0.71 and vs. *CYP27B1* r = 0.77; p < 0.05; **Figure 2E**). In addition, *CYP27B1* expression levels were increased in parallel with *IFNG* and *IL15* mRNAs (r = 0.73 and r = 0.61, respectively; p < 0.05; **Figure 2E**). As in LCL lesions, *IFNG*–*IL15*–*IL32γ* expression levels were positively associated in ML, highlighting the strong association between *IL15* and *IFNG* mRNA levels in both groups of patients. In ML, the expression levels of other pro-inflammatory cytokines *TNF* and *IL6* were also associated with *CYP27B1* and *IFNG*-*IL15*-*IL32γ* expression levels (p < 0.05, **Figure 2E**). It is noticeable that the correlations in ML were stronger than in LCL lesions, except for *VDR* mRNA. In addition, the correlation between *VDR* and *IFNG* mRNA levels was the weakest in ML. Additionally, whereas in LCL *VDR* is strongly associated with *IL32γ* mRNA (r = 0.84, p < 0.001), in ML, it is strongly associated with *IL17* (r = 0.71; p < 0.05; **Figure 2E**). Particularly, the association between the expression levels of *VDR* and *CYP27B1* mRNA occurred only in patients with LCL, and that between *CYP27B1* with *IFNG* was detected only in patients with ML.

### 3.2 Serum levels of vitamin D are increased in patients with cutaneous or mucosal leishmaniasis: association with *IL32γ* expression in mucosal leishmaniasis

**Figure 3:**
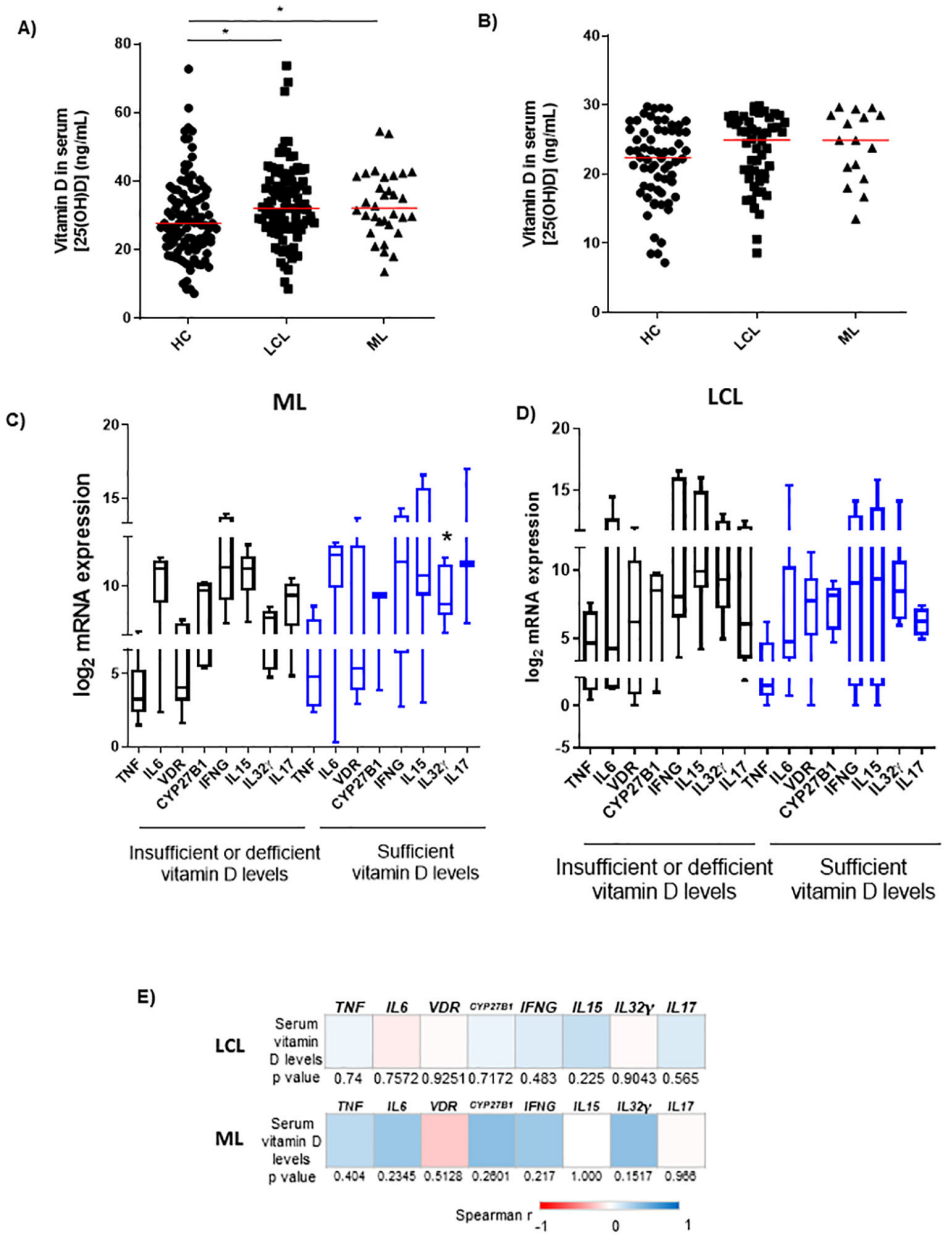
Serum vitamin D levels of healthy controls and patients with cutaneous or mucosal leishmaniasis. **(A)** Serum vitamin D levels of healthy controls (HC; n = 110) and patients with localized cutaneous (LCL; n = 96) or mucosal leishmaniasis (ML; n = 32). *p < 0.05 (Mann–Whitney test). **(B)** Controls and patients with LCL or ML were subdivided into two groups: insufficiency/deficiency (<30 ng/mL) and sufficiency (≥30 ng/mL) of vitamin **(D)** *p < 0.05 compared with controls (Mann–Whitney test). **(C)** mRNA expression of *TNF*, *IL6*, *VDR*, *CYP27B1*, *IFNG*, *IL15*, *IL32γ*, and *IL17* in sufficient *versus* insufficient/deficient serum levels of vitamin D in patients with ML (n = 12); *p < 0.05 (Mann–Whitney test). **(D)** mRNA expression of *TNF*, *IL6*, *VDR*, *CYP27B1*, *IFNG*, *IL15*, *IL32γ*, and *IL17* in sufficient *versus* insufficient/deficient serum levels of vitamin D in patients with LCL (n = 32). **(E)** Heatmaps of correlation between mRNA expression of *TNF*, *IL6*, *VDR*, *CYP27B1*, *IFNG*, *IL15*, *IL32γ*, *IL17*, and serum vitamin D levels of patients with LCL (n = 32) or ML (n = 12); Spearman’s correlation test was used. In panels **(A–D)** data represent individual, median, interquartile range, and minimum/maximum values.

As the expression of the vitamin D pathway-associated genes was increased in ATL lesions, the serum levels of vitamin D [25(OH)D] were measured to evaluate whether circulating vitamin D levels could be associated with the *in situ* immune responses. A high variability of vitamin D concentrations in the serum of controls and LCL patients was detected. The serum vitamin D levels in LCL [median = 33.0 ng/mL (8.6–73.7 ng/mL); n = 82] and ML patients [median = 33.2 ng/mL (18.0–54.5 ng/mL); n = 28] were higher than those of healthy controls [median = 22.7 ng/mL (7.2–72.7 ng/mL); n = 110; p < 0.05; **Figure 3A**]. No difference was found between LCL and ML patients (**Figure 3A**). Furthermore, individuals were subdivided into two groups according to vitamin D levels as sufficient levels (≥30 ng/mL) and insufficient along with deficient levels (<20 ng/mL until 29 ng/mL) (**Figure 3B**). The differences between levels of vitamin D in LCL or ML patients with insufficient/deficient vitamin D levels and those of healthy controls were not statistically significant (**Figure 3B**).

In the lesions of ML patients (n = 12), the levels of *IL32γ* mRNA were higher in the group with sufficiency compared to those with insufficiency/deficiency of vitamin D (**Figure 3C**). The *in situ* gene expression levels were not significantly different between groups separated according to vitamin D levels in LCL patients (n = 32; **Figure 3D**). Despite the increase of *IL32γ* expression in the group of ML with sufficiency of vitamin D, no correlation was detected between serum levels of vitamin D and gene expression levels in mucosal or cutaneous lesions (**Figure 3E**).

### 3.3 The expression of vitamin D-associated genes in leishmaniasis lesions appears to be associated with the clinical or therapeutic outcome

**Figure 4:**
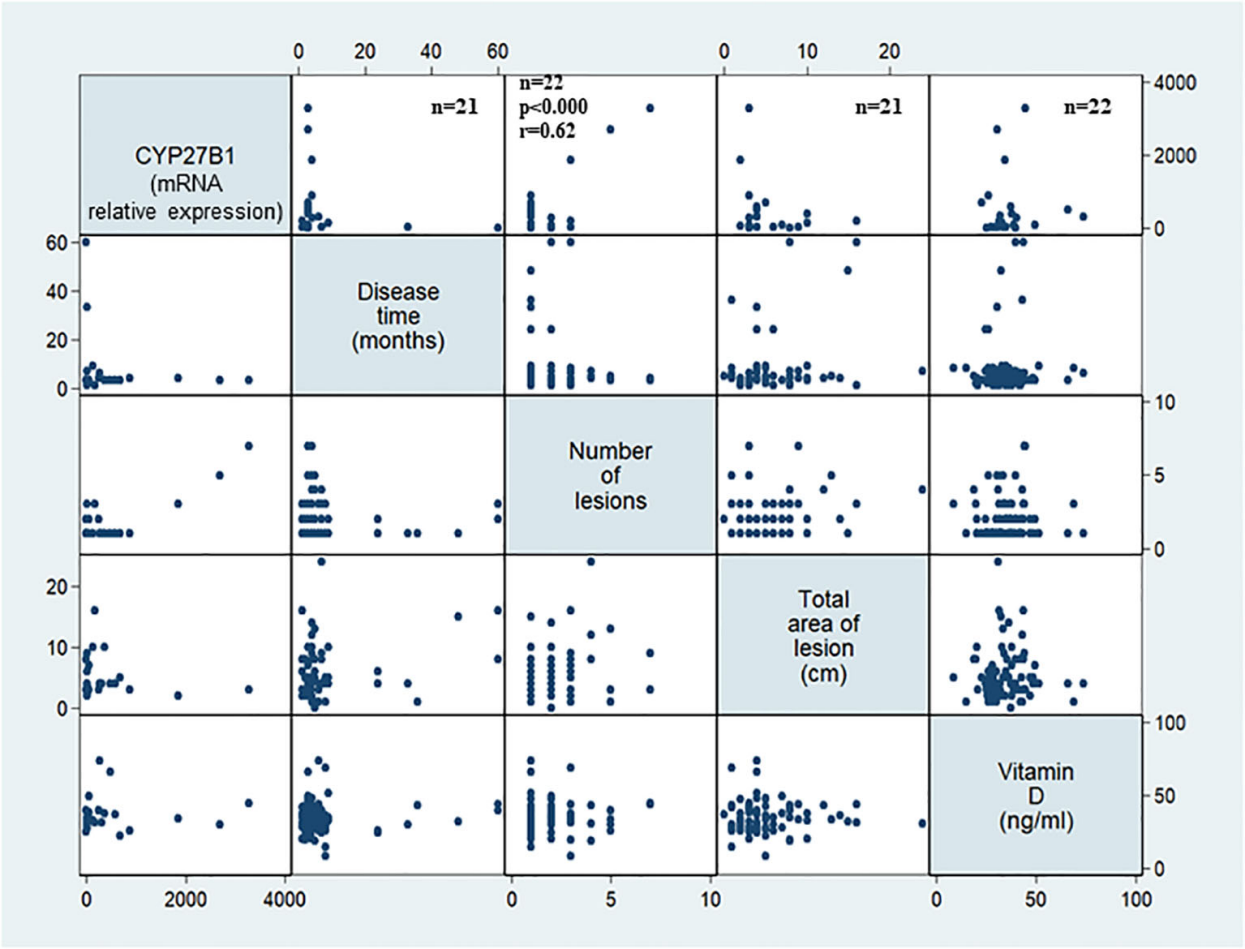
*CYP27B1* expression in association with clinical characteristics of cutaneous localized leishmaniasis patients. The gene expression was evaluated in the lesions of patients with localized cutaneous leishmaniasis (LCL) (n = 22) according to disease duration (months), number of lesions, total area of lesion (cm^2^), and serum levels of vitamin D (ng/mL). Adopted p < 0.05. F test.

In order to investigate whether the expression of vitamin D pathway-associated genes is correlated with the clinical outcome, duration of the disease, number, and total area of lesions were evaluated. The disease duration was 1 to 60 months in patients with LCL and 2 to 600 months in patients with ML. In patients with LCL, the number of lesions was positively correlated with the *CYP27B1* mRNA levels. The mRNA expression levels of other genes evaluated were not significantly associated with disease duration, size, or number of lesions at the diagnosis of LCL or ML (**Table 1**, **Figure 4**).

**Table 1:** Expression of genes evaluated in lesions of patients with cutaneous leishmaniasis in relation to the number of lesions.

Number of lesions*					
Dependent variable	R^2^	R^2^-adjusted	SD	F	p**
*TNF*	0.0507	0.0112	249.872	1.28	0.2687
*IL6*	0.0242	−0.0223	9,952.007	0.52	0.4786
*VDR*	0.1097	0.0754	1,707.49	3.2	0.0852
***CYP27B1***	**0.6251**	**0.6063**	**895.2825**	**33.35**	**<0.0001**
*IFNG*	0.0258	−0.009	20,817.95	0.74	0.3968
*IL15*	0.0258	−0.009	17,333.55	0.74	0.3965
*IL32γ*	0	−0.0357	3,972.831	0	0.9993
*IL17*	0.0224	−0.0387	1,327.71	0.37	0.5533

SD, standard deviation; F, F test of Levene.

*n = 154 patients with localized cutaneous leishmaniasis (LCL).

** Values with p < 0.05 considered statistically significant. *Values with p < 0.05 considered statistically significant are represented by bold font.

The patients were treated with antimonial, liposomal amphotericin B, or other drugs and were followed up for several months. In the group of patients with ML, the mRNA levels for all genes evaluated did not significantly differ between treated patients with clinical cure until 90 days and those with therapeutic failure (cure or not after 90 days). However, the group of patients with LCL that presented therapeutic failure showed higher *VDR* expression levels than the group with clinical cure (**Table 2**).

**Table 2:** Expression of genes evaluated in lesions of patients with cutaneous leishmaniasis in relation to the therapeutic outcome.

Therapeutic outcome*						
Dependent variable	N	Mean	SD	CI (95%)		p**
*TNF*	1 - Cure	44.20472	65.5375	6.3645	82.04491	0.1093
	2 - Failure	204.48	474.442	−234.4013	643.271	
*IL6*	1 - Cure	5,211.823	13,026.97	−2,660.295	13,083.94	0.7297
	2 - Failure	1,466.765	1,354.375	−2,293.582	5,227.112	
***VDR***	**1 - Cure**	**279.5098**	**374.2918**	**63.4001**	**495.6194**	**0.0109**
	**2 - Failure**	**1,704.084**	**2,122.594**	**−258.987**	**3,667.156**	
*CYP27B1*	1 - Cure	459.27	946.4521	−176.57	1,065.1	0.3304
	2 - Failure	676.08	758.7734	−266.06	1,618.22	
*IFNG*	1 - Cure	8,278.562	23,810.24	−4,409.02	20,966.14	0.618
	2 - Failure	5,454.56	7,243.63	−1,244.68	12,153.8	
*IL15*	1 - Cure	9,302.801	16,830.42	334.5035	18,271.1	0.6487
	2 - Failure	6,581.777	11,599.18	−4,145.678	17,309.23	
*IL32γ*	1 - Cure	2,095.676	4,486.649	−295.0907	4,486.442	0.4262
	2 - Failure	2,464.574	3,876.246	−1,120.356	6,049.504	
*IL17*	1 – Cure	99.6296	44.8127	67.5725	131.6867	0.4254
	2 - Failure	111.753	198.5653	−204.2082	427.7154	

Note. SD, standard deviation; CI, confidence interval.

*Cure, n = 47; failure, n = 18.

** Values with p < 0.05 considered statistically significant. Friedman test. *Values with p < 0.05 considered statistically significant are represented by bold font.

### 3.4 Single-nucleotide polymorphisms in genes of vitamin D pathway in cutaneous and mucosal leishmaniasis

In this study, we evaluated the distribution of *IL32* rs4349147, *IL32* rs1555001, and *IL32* rs2239303 as well as SNPs in *VDR* (rs7975232; rs2248098), *CYP27B1* (rs4646536), and *IL15* (rs10519613; rs3775597) in patients with ATL and HC. The frequency of genotypes and alleles are shown in **Supplementary Table S6**. A lower frequency of allele A of *IL32* rs1555001 was detected in patients with ML than in HC (OR = 0.56, 95% CI = 0.34–0.92; **Table 3**).

**Table 3:** Analyses of single-nucleotide polymorphisms in healthy controls and patients with cutaneous or mucosal leishmaniasis.

	LCL × HC		ML × HC	
SNP	OR	CI 95%	OR	CI 95%
*IL32*rs1555001 T>A	1.0833	0.7184–1.6336	**0.5629**	0.3415–0.9276*
*VDR*rs7975232 C>A	1.2053	0.8441–1.7211	1.1278	0.7086–1.7950
*VDR*rs2248098 A>G	0.9524	0.6737–1.3464	1.1143	0.7031–1.7661
*IL32*rs2239303 G>A	1.1482	0.7860–1.6773	0.7518	0.4658–1.2135
*IL32*rs4349147 A>G	1.032	0.6876–1.5491	0.6649	0.4017–1.1007
*IL15*rs10519613 C>A	0.8572	0.5210–1.4103	1.4224	0.7886–2.5656
*IL15*rs3775597 A>G	0.813	0.5120–1.2909	1.3116	0.7521–2.2872
*CYP27B1* rs4646536 *A>G*	0.9087	0.6142–1.3446	0.9284	0.5521–1.5612

OR, odds ratio; CI, confidence interval; HC, healthy controls (n = 110); LCL, localized cutaneous leishmaniasis (n = 161); ML, mucosal leishmaniasis (n = 59).

*Values with p < 0.05 considered statistically significant. *Values with p < 0.05 considered statistically significant are represented by bold font.

### 3.5 Single-nucleotide polymorphism in VDR gene associated with therapeutic outcomes in mucosal leishmaniasis and with expression of *IL6* mRNA in lesions of patients with cutaneous leishmaniasis

The selected SNPs were analyzed in groups of patients according to their clinical characteristics, vitamin D levels, and therapeutic outcomes (**Supplementary Tables S6**, **S7**, **S8**). None of the allelic distributions of the SNPs evaluated showed a statistically significant association with the clinical characteristics of patients with ATL as well as with the levels of vitamin D (**Supplementary Tables S6**, **S7**). Only among patients with ML was it observed that carriers of allele A of the VDR rs7975232 SNP had a higher chance of therapeutic failure (**Table 4**; **Supplementary Table S8**).

**Table 4:** Analyses of single-nucleotide polymorphisms in patients with mucosal leishmaniasis according to therapeutic outcomes.

ML: clinic cure			ML: failure		OR allele 2	
SNP	Frequency of allele 1	Frequency of allele 2	Frequency of allele 1	Frequency of allele 2	OR	CI 95%
*IL32*rs1555001 T>A	0.6154	0.3846	0.6667	0.3333	0.8	0.2272–2.8170
*VDR*rs7975232 C>A	0.6538	0.3462	0.25	0.75	**5.6667**	1.4108–22.7611*
*VDR*rs2248098 A>G	0.6154	0.3846	0.3125	0.6875	3.52	0.9405−13.1739
*IL32*rs2239303 G>A	0.4231	0.5769	0.5556	0.4444	0.5867	0.1745–1.9719
*IL32*rs4349147 A>G	0.3846	0.6154	0.3333	0.6667	1.25	0.3550–4.4016
*IL15*rs10519613 C>A	0.7692	0.2308	0.9444	0.0556	0.1961	0.0214–1.7938
*IL15*rs3775597 A>G	0.7308	0.2692	0.9444	0.0556	1.1597	0.0178–1.4340
*CYP27B1 rs4646536 A>G*	0.7308	0.2692	0.6875	0.3125	1.2338	0.3145–4.8405

OR, odds ratio; CI, confidence interval; ML, mucosal leishmaniasis (n = 59).

*Values with p < 0.05 considered statistically significant are represented by bold font.

**Figure 5:**
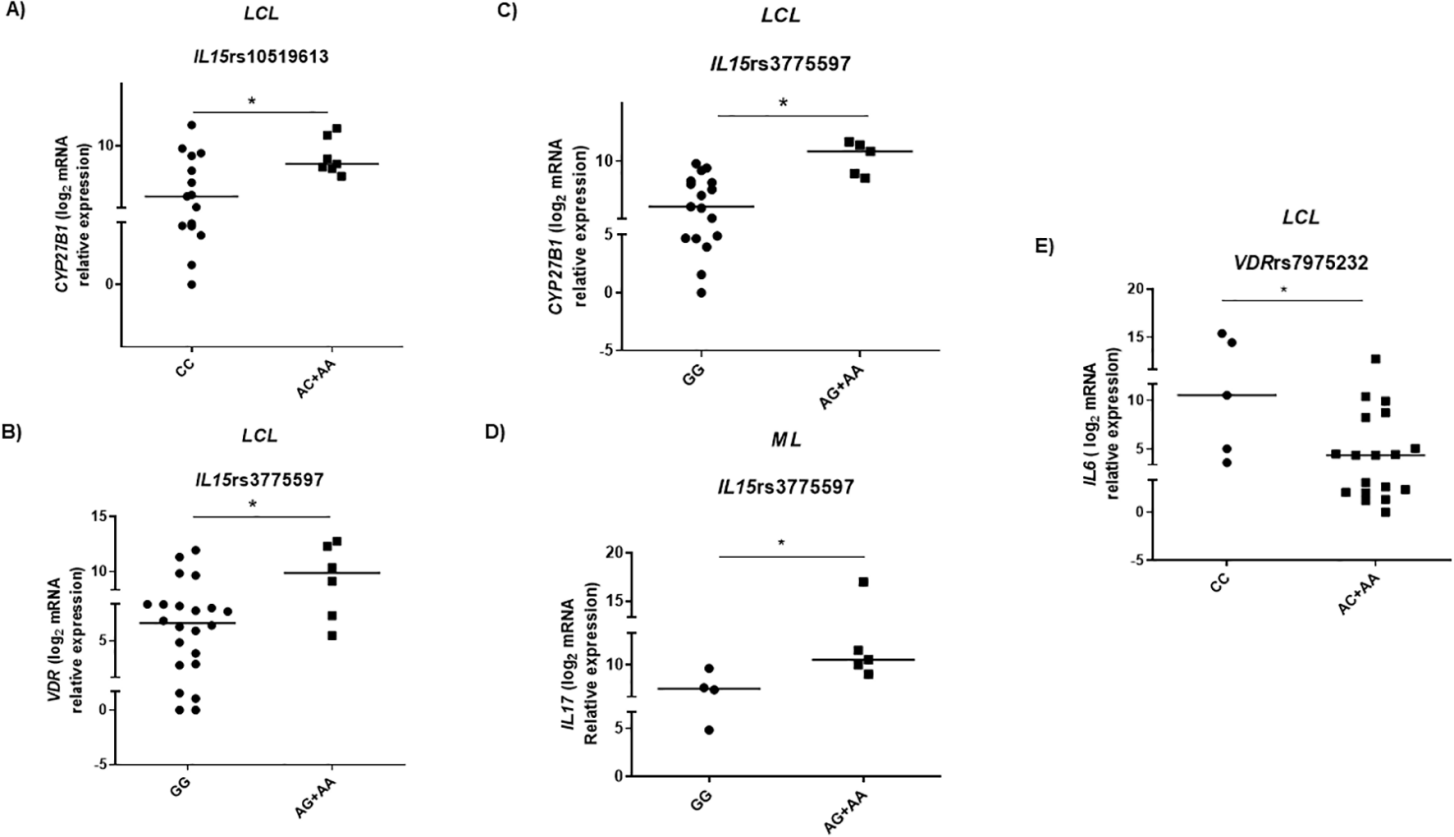
Gene expression in American tegumentary leishmaniasis patients according to genotypes of single-nucleotide polymorphisms in genes associated with vitamin D pathway. **(A)** *CYP27B1* mRNA levels according to *IL15* SNP rs10519613 genotypes under dominant model, patients with localized cutaneous leishmaniasis (LCL) (CC = 15; AC+AA = 7); **(B)** *VDR* (GG = 22; AG+AA = 6) and **(C)** *CYP27B1* (GG = 17; AG+AA = 5) expression in lesions of patients with LCL; **(D)** *IL17* expression in lesions of mucosal leishmaniasis (ML) patients according to *IL15* SNP rs3775597 genotypes under recessive model (GG = 4; AG+AA = 5). **(E)** *IL6* mRNA levels distributed according to *VDR* SNP rs7975232 genotypes in ML (CC = 5; AC+AA = 18). The data are presented as median. Adopted *p < 0.05 (Mann–Whitney test).

We adopted recessive, dominant, and additive genetic models using logistic regression to analyze the association between genotype frequencies of variants and gene expression levels. The mRNA expression levels in the lesions were differentially distributed between the selected SNP genotypes under the recessive model. Although rs1555001 SNP in *IL32* was differentially distributed in groups of ML patients and HC, no association was detected between the genotypes of this SNP and levels of *IL32* mRNA in the lesions. In the patients with LCL carriers of *IL15* SNP rs10519613 AC and AA genotypes (carriers of altered allele A), the *CYP27B1* expression was higher than in those with CC genotype (**Figure 5A**). In addition, those LCL patients with GG genotype of *IL15* rs3775597 (homozygous for altered allele G) presented lower *VDR* and *CYP27B1* expressions in the lesions than those with AG and AA genotypes (**Figures 5B, C**). Patients with ML presented lower levels of *IL17* mRNA when carrying the GG genotype than those with AG or AA genotypes of *IL15* rs3775597 (**Figure 5D**). Concerning *VDR* SNP, although no significant differences were observed in frequencies of this SNP in patients with ML, LCL, or HC, among LCL patients, those with AC or AA genotypes of *VDR* SNP rs7975232 (carriers of altered allele A) presented lower *IL6* expression in the lesions than those with CC genotype (**Figure 5E**).

**Figure 6:**
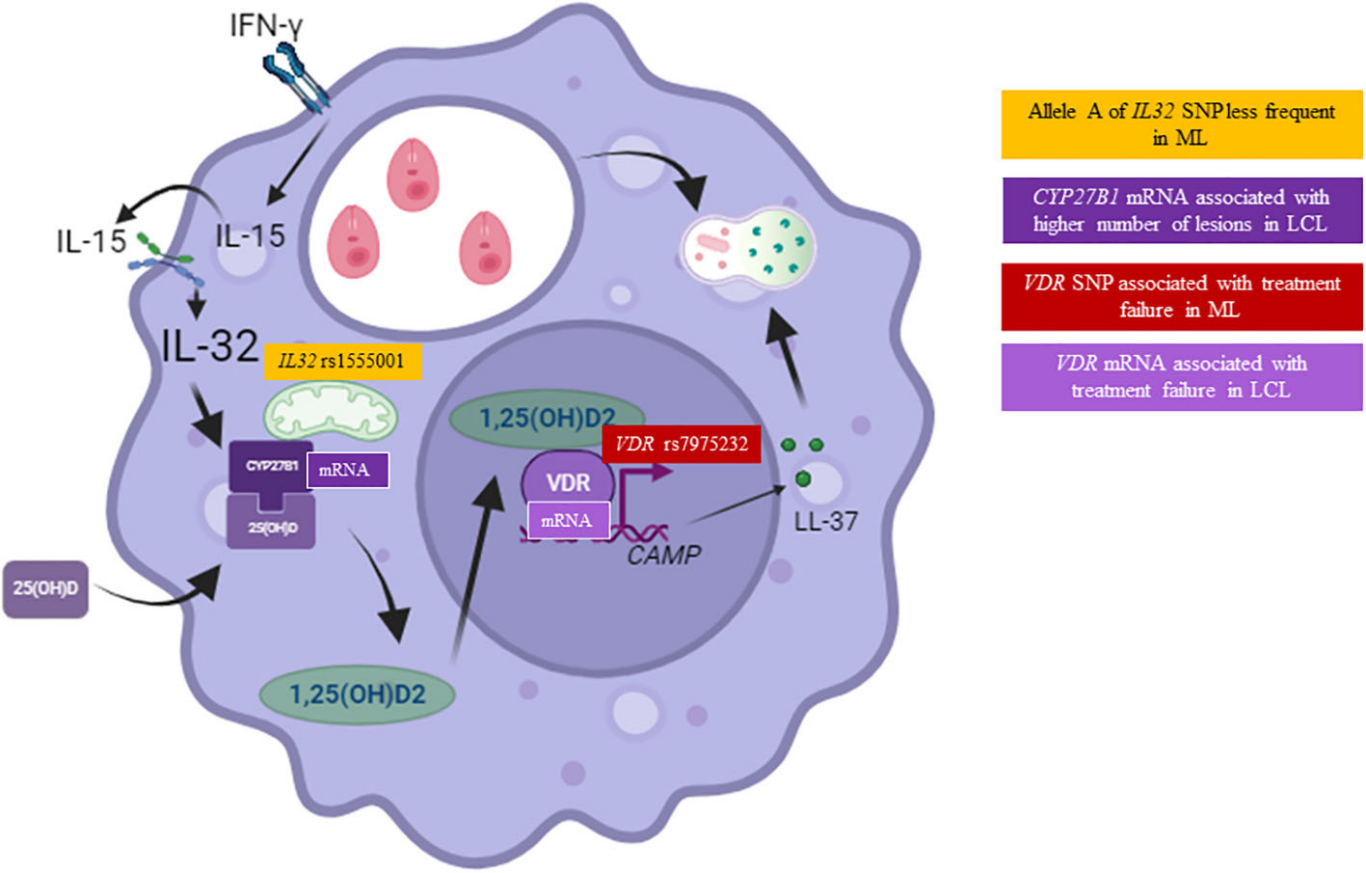
Single-nucleotide polymorphisms and expression of genes related to vitamin D pathway associated with American tegumentary leishmaniasis (ATL). Carriers of the altered allele A of the *IL32* SNP rs1555001 were less likely to be in the mucosal leishmaniasis (ML) group (orange square). The levels of *CYP27B1* mRNA relative expression were associated with the number of lesions in patients with localized cutaneous leishmaniasis (LCL) (dark purple square). Carriers of the altered allele A of the *VDR* SNP rs7975232 were more likely to be in the treatment failure group of ML patients (red square). Finally, levels of *VDR* mRNA relative expression were higher in the group of LCL patients who experienced treatment failure (light purple square).

## 4 Discussion

To investigate a possible role of vitamin D in susceptibility or resistance to ATL, the present study searches for the expression of pro-inflammatory cytokines and components of the vitamin D pathway in lesions of patients with ATL in a less severe clinical form, LCL, and in the most severe form, ML. Further, SNPs in genes of two components of the vitamin D pathway (CYP27B1 and VDR) as well as in two cytokines related to this pathway (IL-15 and IL-32) were evaluated in cohorts of healthy individuals and ATL patients with LCL or ML. It was shown that in ML lesions, *IL6*, *IFNG*, and *IL17* mRNA levels were higher than in LCL lesions, suggesting the involvement of these cytokines in the immunopathogenesis of ATL. The pro-inflammatory cytokines IL-6 and IL-17 have been found in ML lesions with necrotic areas infiltrated by neutrophils (Boaventura et al., 2010). The number of CD4^+^IL-17^+^ cells in ML lesions is higher than in CL (Bacellar et al., 2009). However, IL-17 also appears to participate in the pathogenesis of CL, contributing to the inflammatory infiltration at the site of infection (Bacellar et al., 2009). In tissue lesions of CL patients infected with *L. tropica* (Katara et al., 2013), high levels of Th17 lymphocyte markers were found. Together, data suggest that IL-6 and IL-17 can increase the inflammatory response contributing to tissue damage in ML and LCL patients, but with predominance in ML. These results are in agreement with our data showing a positive correlation between mRNA *IL17* and *IL6* levels in ML stronger than in LCL lesions.

The higher expression of *IFNG* in ML than in LCL lesions detected in the present study reflects the strong Th1 immune responses in ML caused by *L*. (*V*.) *braziliensis* (Bacellar et al., 2002; Silveira et al., 2009), and it is in accordance with the higher number of IFNγ-expressing cells detected in ML than in CL lesions (Faria et al., 2005). It is known that IFNγ and TNF-α, markers of Th1 response, are relevant cytokines to improve the macrophage leishmanicidal activity (Liew et al., 1990) but at the same time participate in tissue lesion (Bacellar et al., 2002). In our study, *TNF* mRNA was detected in low levels in both LCL and ML lesions, but it was positively correlated with *IFNG*, *IL17*, and *IL6* more strongly in ML lesions than in LCL lesions. Altogether, the results strengthen the higher inflammatory profile detected in ML than in LCL.

The IL-32 is a pro-inflammatory cytokine known to induce *CYP27B1* and *VDR* expressions, and this cytokine is part of a network with IFNγ and IL-15 to induce defense mechanisms against microorganisms (Montoya et al., 2014; Silva et al., 2020). In the current study, a stronger association between *IFNG* and *IL15* mRNA levels was detected in LCL than in ML lesions. As these cytokines induce IL-32 production (Montoya et al., 2014), it was expected that *IL32G* mRNA levels were, in turn, associated with these cytokines. In fact, *IL32G* mRNA levels were weakly correlated with *IFNG* and *IL15* mRNA levels in both types of lesions but were highly associated with *IL15* expression, especially in ML than in LCL lesions. Both IL-15 and IFNγ are inducers of IL32γ in human macrophages, and they were strongly correlated in LCL as well as ML lesions. The axis IFNγ/IL-15/IL32γ is very important to the vitamin D-dependent microbicidal activity of human macrophages (Montoya et al., 2024; Silva et al., 2020). Concerning the mRNA levels of the components of the vitamin D pathway, *VDR* and *CYP27B1* were associated with LCL lesions. We found a strong association between *IL32G* and *VDR* as well as *IL17* and *VDR* expression levels in LCL and ML lesions, respectively. The data suggest that in mild leishmaniasis LCL, the lesions present a less inflammatory microenvironment with control of the parasites. However, patients with ML presented a strong positive correlation between *IL32G* and *TNF*, *IL17*, and *IL6*, thus indicating the participation of IL32γ in the inflammatory process. These results are in accordance with our previous results, where the association between TNF and IL32γ was shown only in patients with ML (Galdino et al., 2014). Together, these data suggest that *IL-32/CYP27B1/VDR* expression is related to microbicidal vitamin D-dependent mechanisms in LCL, contributing to protection against the parasites. Antimicrobial peptides such as cathelicidin and β-defensin-2, which are induced by active vitamin D in M1 macrophages, contribute to microorganism elimination (Montoya et al., 2014; Crauwels et al., 2019). However, in severe leishmaniasis, ML, IL32γ is highly associated with an inflammatory profile, which can be responsible for immunopathogenesis.

The link between vitamin D and IL32γ in ML is strengthened by the finding that the patients with sufficient serum levels of vitamin D expressed high levels of *IL32G* mRNA in the lesions, suggesting that vitamin D could increase IL32γ production. How vitamin D acts to control the expression of *IL32* is not known and deserves further investigation. In the current study, IL32γ was associated with all pro-inflammatory cytokines evaluated in mucosal lesions, including IL-15. The cytokine IL-15, strongly associated with IL32γ in ML, is a pro-inflammatory cytokine that can control the growth and proliferation of T lymphocytes and NK cells as well as macrophage differentiation in M1/microbicidal profile, crucial for controlling intracellular microorganisms; however, it is also associated with the pathogenesis of chronic immune-mediated diseases (Waldmann, 2014; Yang et al., 2015). Few studies have evaluated IL-15 in leishmaniasis. In visceral leishmaniasis, it was shown that IL-15 is produced during the infection caused by *Leishmania infantum* (Milano et al., 2002) and increases the killing of this parasite (D’agostino et al., 2004; Oliveira et al., 2015). The cytokine IL-15 is also produced by monocytes cultured with New World *Leishmania* spp. antigens (Carrada et al., 2007; Lago et al., 2018), but its biological function was not evaluated in these studies. That IL-15 can be associated with parasite control is strengthened by the strong correlation between the *IL15* and *IFNG* mRNA levels in LCL detected in our study. However, in addition to known cytokines involved in the immunopathogenesis of ATL, such as IFNγ, TNF, and IL32γ, our present data suggest that IL-15 should be especially considered in ML.

The expression of cytokines at the diagnosis time could be a marker of prognosis in ATL; however, despite high levels of *IFNG*, *IL15*, and *IL32G* mRNA in the early LCL lesions, these levels could not be associated with the therapeutic outcome in the present study. We detected 27.7% and 41.0% of therapeutic failure in LCL and ML patients, respectively, a lower rate than previously reported in Brazil for LCL (Carvalho et al., 2022), but in the same range for ML (30%–90% depending on the geographic area; Goto and Lindoso, 2012). Surprisingly, we found that the *VDR* mRNA levels in patients with LCL were higher in the failure outcome group. As the VDR mRNA levels are not only expressed in macrophages, where they are relevant for microbicidal activity, the increased levels of VDR in other cells present in the lesions can explain these results. It is known that vitamin D inhibits T helper lymphocyte cytokines, including Th1-derived cytokines required for control of *Leishmania* ssp. Even in macrophages, although vitamin D stimulates microbicidal activity, it inhibits pro-inflammatory cytokine production. In addition, vitamin D increases the production of anti-inflammatory/immunosuppressive cytokines, which were not evaluated in the present study (Ghaseminejad-Raeini et al., 2023). Thus, in the lesions, the immunomodulatory effects mediated by vitamin D–VDR on different immune cells can interfere with the outcome of the disease.

We also evaluated the SNPs in genes of the vitamin D pathway. No study has yet investigated SNPs in the *IL15* gene in association with ATL outcomes. We found that allele A of *IL15* rs10519613 was associated with high expression of *CYP27B1*. It was not associated with serum vitamin D levels nor with the therapeutic outcome of LCL. Although not in infectious diseases, there is a report of the association between the AA genotype of this SNP and hyperdiploidy, a clinical feature in acute lymphoblastic leukemia (ALL) (Rots et al., 2018), and allele A was associated with the risk of minimal residual disease–positivity (indicator for ALL) (Dawidowska et al., 2016). The allele A of this *IL15* SNP was also correlated with the risk of type 1 diabetes (Zouidi et al., 2014). The *IL15* rs10519613 is located at exon 6, 83 bp behind the stop codon, and the AA+AC genotypes and the allele A of this SNP were found as risk factors for coronary heart disease. Furthermore, the AC genotype and allele A were associated with high levels of IL-15 in acute form, and all subjects evaluated with the CC genotype presented lower levels of IL-15 (Gokkusu et al., 2010). Another genetic variation of the *IL15* gene, the *IL15* rs3775597, was selected in our evaluation due to the relevant frequency of the G variant allele in the American population, but there is yet no study that has investigated it in infectious or non-infectious disease outcomes. Only one report evaluated this SNP as a risk factor for hypertension but did not achieve conclusive results (Taylor et al., 2010). We demonstrated that in LCL, the GG *IL15* rs3775597 genotype was associated with low *VDR* and *CYP27B1* expressions. There was no association with vitamin D levels or with treatment outcomes in carriers of this SNP *IL15*. Despite this, we detected two variants of *IL15* that were differentially associated with CYP27B1 expression. As IL-15 induces CYP27B1, in an IL-32-dependent manner (Montoya et al., 2014), these results suggest that the *IL15* SNP rs10519613 allele A can be associated with a gain of function increasing CYP27B1 and favoring the control of the parasites in macrophages of LCL patients. However, *IL15* SNP rs10519613 can interfere with the capacity of IL-15 to induce CYP27B1. In any situation, the data highlight a possible role of IL-15 in LCL. Concerning ML, the GG genotype of the *IL15* rs3775597 variant was associated with low *IL17* expression. This SNP is located in an intronic region, so the influence on cytokine production needs to be further investigated to better understand the mechanism by which this SNP can decrease IL17 expression, or whether this is an indirect effect, depending on other cytokines in ML lesions.

We had previously studied the IL32 rs1555001, but no significant association with ATL manifestations was found (Dos Santos et al., 2020) as well as with acute lung injury (Arcaroli et al., 2011) or with carotid artery calcified plaque in type 2 diabetes mellitus (Lehtinen et al., 2011). Now, with more patients in the cohort, we found that allele A was significantly associated with HC, which means that the risk of carriers of allele A not present in ML is significant. Thus, this can associate this SNP with mechanisms of resistance to develop ML. Previously, we showed that carriers of the AA genotype presented low production of IFNγ and IL-22 in cultures of peripheral blood mononuclear cells stimulated with *L. braziliensis* (Dos Santos et al., 2020). It is known that IL32γ and IFNγ play a role in infection control, but they exacerbate the responses linked to tissue damage present in ML (Ribeiro-De-Jesus et al., 1998; Oliveira et al., 2011; Galdino et al., 2014). As our previous data suggested, the genetic variant *IL32* rs1555001 can reduce the production of IL32γ and IFNγ, and this can promote an environment that is less inflammatory and thus contribute to the resistance of ML development. However, IL-22 was found to be associated with control of inflammation and wound healing, but in a leishmaniasis model, this appears to be dependent on the level of tissue damage (Gimblet et al., 2015). The production of these immunomodulators, especially IL-22, in association with this *IL32* genetic variation, needs to be further investigated.

Only two previous studies have investigated genetic variants related to vitamin D in association with leishmaniasis. One of them found no significant association between SNPs in the VDR gene with increased risk of *L. tropica* infection (Shabandoust et al., 2020), but another one demonstrated that the allele A of *VDR* rs7975232 (*Apa*I) was associated with susceptibility to CL in Saudi patients (Salem et al., 2023). We found that the presence of this allele was associated with low IL6 expression in the cutaneous lesions. Moreover, we reported in this study that the carriers of allele A with ML have more probability of therapeutic failure. The *Apa*I SNP, located in the intron 8 of the *VDR*, presents the C reference allele that changes to A and does not change the amino acid sequence of the VDR protein. However, the SNP could affect mRNA stability and *VDR* expression by LD (Mahto et al., 2018; Triantos et al., 2018). If *Taq*I (*VDR rs731236*) is in high LD with *Apa*I, its functional effect is the possible modification of one of the zinc fingers of the nuclear signaling heterodimer that binds to the VDREs located in the target genes (Mahto et al., 2018). We can speculate that possibly that in patients with ML carriers of the A variant allele, there is an LD between *Apa*I and another *VDR* SNP that compromises the binding of VDR to VDREs in DNA and, consequently, the production of antimicrobial peptides, favoring the parasite persistence and the therapeutic failure. In addition, as commented above, less stimulation of VDR can lead to high inflammation in lesions due to the missing control of pro-inflammatory cytokines by vitamin D in T lymphocytes (Ghaseminejad-Raeini et al., 2023). Previous data about this VDR SNP in association with treatment outcomes in other infectious diseases showed that the allele A of *Apa*I was considered the pretreatment genetic predictor of sustained hepatitis B surface antigen (HBsAg) and loss in hepatitis B early antigen (HBeAg) in patients with chronic hepatitis B with pegylated interferon (Peg-IFN) monotherapy (Shan et al., 2019). Furthermore, the VDR haplotype composed of the C allele of *Apa*I was a predictor of pegylated-interferon/ribavirin-based therapy failure in chronic hepatitis C (CHC) Caucasian patients (Baur et al., 2012; García-Martín et al., 2013), but not in CHC Asian patients (Hung et al., 2016). These data pointed out that, in contrast to the impact on treatment outcomes in hepatitis B and C, the altered allele A of *Apa*I could be a genetic marker of therapeutic failure in ML.

It is very difficult to reconcile all the results obtained in the current study, mainly those of mRNA expression versus SNPs and those with clinical/therapeutic outcomes. We confirmed that ML presents lesions with a stronger inflammatory profile than lesions of patients with LCL. In addition, we also confirmed our previous results about the strong connection between IL 32γ and TNF in ML and the role of vitamin D microbicidal pathway dependent on IL32γ and IL-15 in human macrophages. Here, the results suggest that for LCL, the axis IL32γ/IL-15/CYP27B1/VDR, although very important to control the parasites in human macrophages, can be affected by the effects of vitamin D on T lymphocyte cytokines in the lesions. This can be illustrated by two different *IL15* SNPs that differentially affect the vitamin D pathway gene expression, *CYP27B1* and *VDR*, whose expressions were associated with the high number of lesions and therapeutic failure, respectively. In addition, in ML, the axis IL32γ/TNF/IL-15/IFNγ/IL-6/IL-17 is highly inflammatory in the immunopathogenesis of the disease. In this pro-inflammatory environment, the VDR SNP was associated with therapeutic failure, which may be associated with the difficulty of vitamin D in controlling the inflammatory cytokines (**Figure 6**). This type of study is relevant to identify possible players and their connection to investigate their roles in ATL. A low number of studies on vitamin D, IL-32, and IL-15 have been published, and one of the difficulties is that the axis IL-15/IL32γ/vitamin D cannot be evaluated in a mouse model. Mouse does not present IL-32, and the β-defensin and cathelicidin genes are not controlled by vitamin D in these animals as in human beings. In addition, the IL-32 receptor was not yet identified. Together, these points can highlight the relevance of genetic studies on the cytokine/vitamin D pathway in ATL and other diseases.

## Data Availability

We are in according to make this material available upon request to interested researchers.
