# Supplementary material for: Single-nucleotide polymorphisms in genes associated with the vitamin D pathway related to clinical and therapeutic outcomes of American tegumentary leishmaniasis
Source: Front Cell Infect Microbiol. 2025 Jan 8;14:1487255. doi: 10.3389/fcimb.2024.1487255 (PMC11750871; doi:10.3389/fcimb.2024.1487255)
Supplement: Supplementary file 1 [file Table1.docx]

Supplementary Material


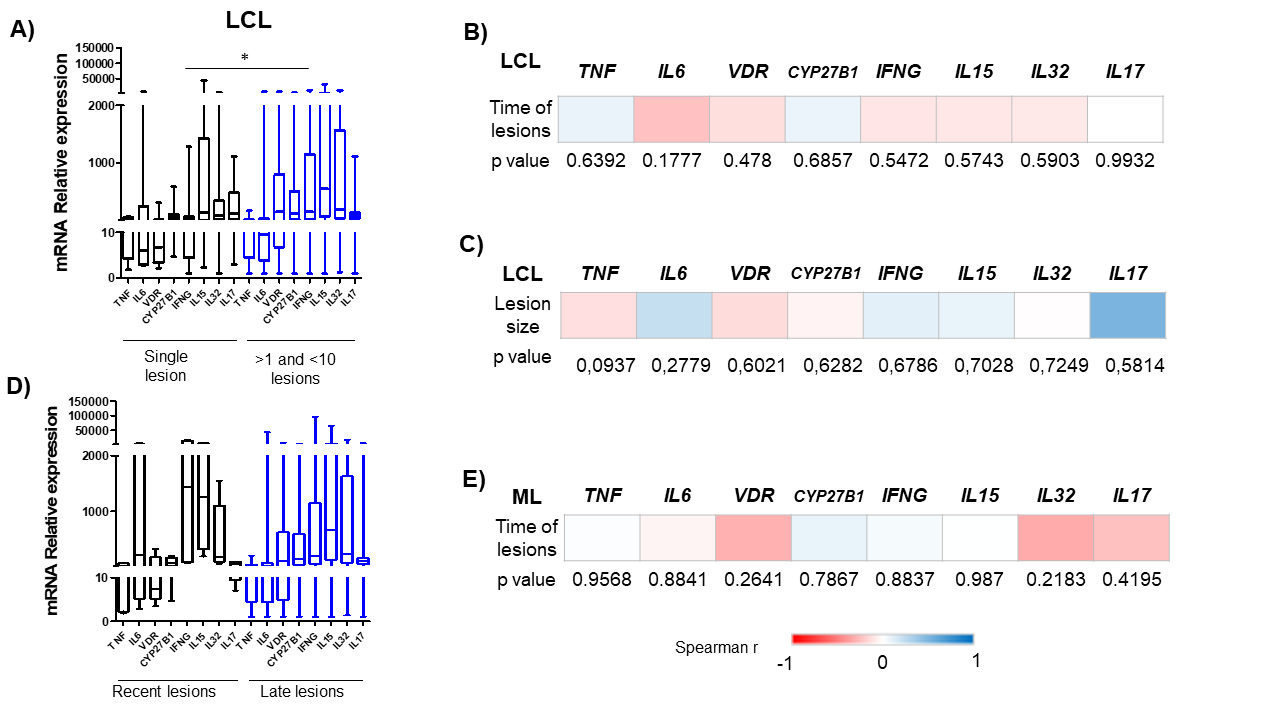


**Supplementary Figure 1.** Gene expression in association with clinical and therapeutic outcomes of leishmaniasis patients. **(A)** Gene expression in groups of LCL patients with different number of lesions. *p < 0.05 (Mann-Whitney test). **(B)** Correlation between the gene expression and the time of lesions (n=44), and **(C)** single lesion size (n = 29) of LCL patients. **(D)** Gene expression in groups of LCL patients with different stage of disease: recent lesions ( ≤ 1 month) or late lesions (> 1 month). **(E)** Correlation between the gene expression and the time of lesions (n=18) of ML patients. Spearman correlation´s test with significance level p < 0.05.


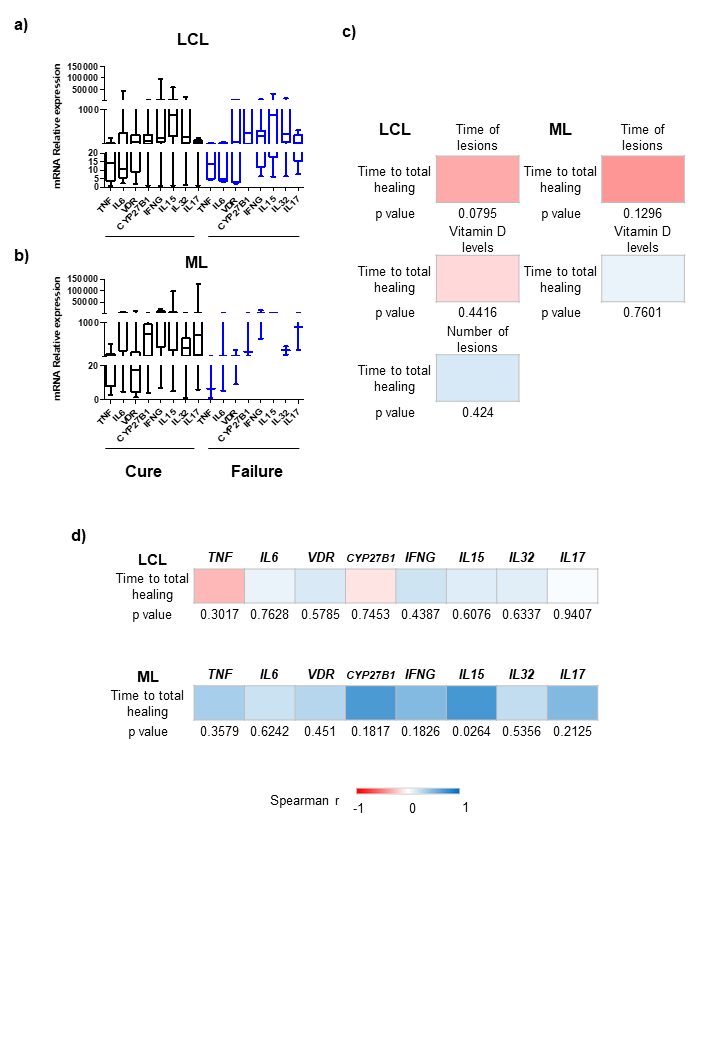


**Supplementary Figure 2.** Gene expression in association with therapeutic outcomes of leishmaniasis patients. **(a)** Gene expression in groups with cure (complete cicatrization up 90 days) or failure (complete cicatrization or not after 90 days) among LCL and **(b)** ML patients. **(c)** Correlation between the clinical parameters of LCL and ML patients with time to total healing. **(d)** Correlation between the gene expression of LCL and ML patients with time to total healing. The time to healing consists in time required after the end of the first treatment schedule until the total re-epithelialization of the lesions. This time ranged from one to six months. Spearman correlation´s test with significance level p < 0.05.

**Supplementary Table 1.** Characteristics of patients with American Tegumentary Leishmaniasis in the group for gene expression evaluation in the lesions (n = 62)


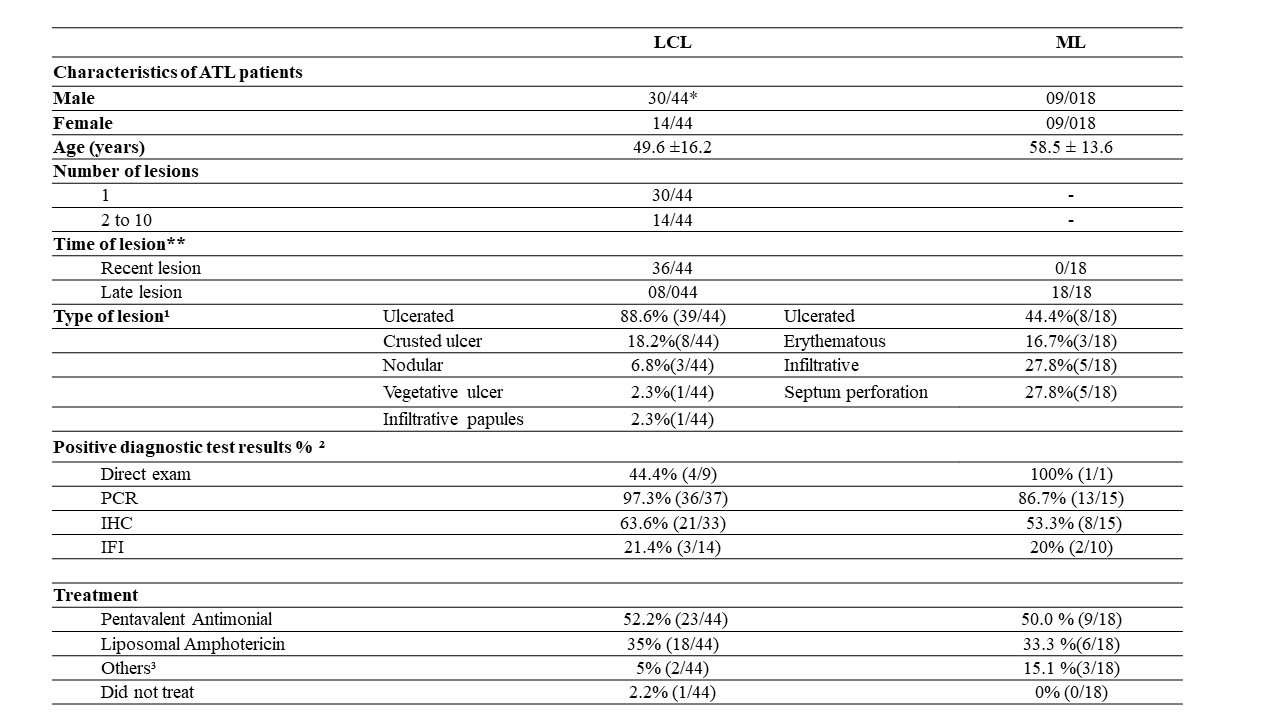


LCL= Localized Cutaneous Leishmaniasis; ML= Mucosal Leishmaniasis; PCR= Polymerase Chain Reaction; IHC= Immunohistochimistry; IFI= Indirect Immunofluorescence

*n/total

**Recent lesion = time of disease lesser than or equal to one month; Late lesion=time of disease higher than one month. The fragments of lesions were obtained from patients in Hospital de Doenças Tropicais Dr Anuar Auad, Goiânia, Goiás.

¹ There were patients with more than one type of lesion, then the sum is expected to be more than 100%.

² positive/total tested

³Fluconazol or Itraconazol.

**Supplementary Table 2.** Characteristics of control group (n = 110) and patients with American Tegumentary Leishmaniasis (n = 220) with active or not active lesions for genetic evaluation


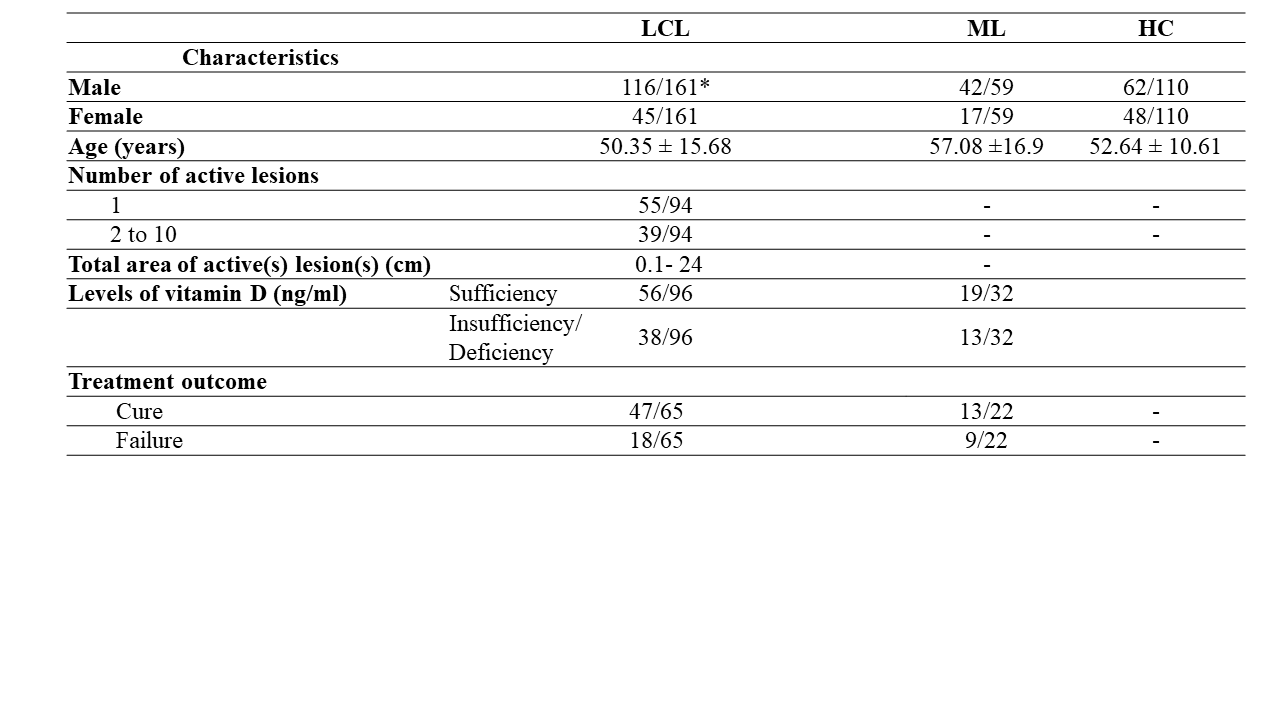


HC= Healthy Controls; LCL= Localized Cutaneous Leishmaniasis; ML= Mucosal Leishmaniasis;

*n/total

**Supplementary Table 3.** Primers sequence used in the qPCR assay.


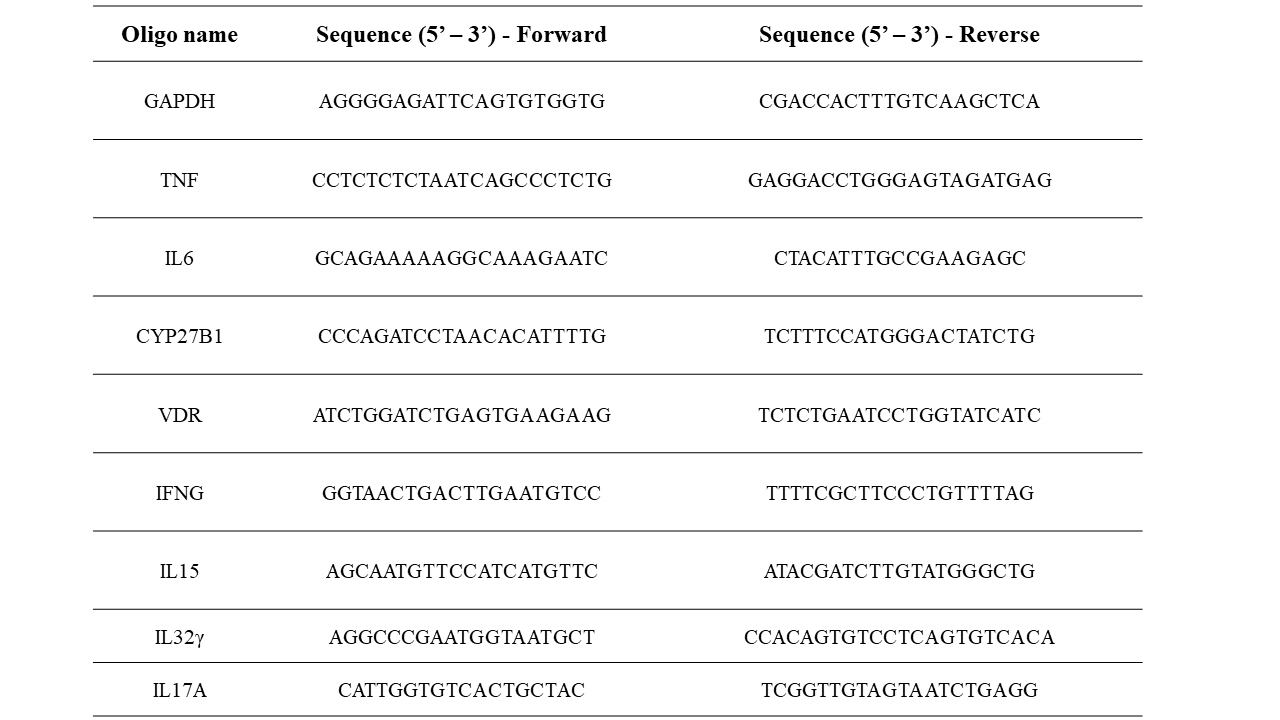


**Supplementary Table 4.** SNP information for TaqMan PCR assay.

**
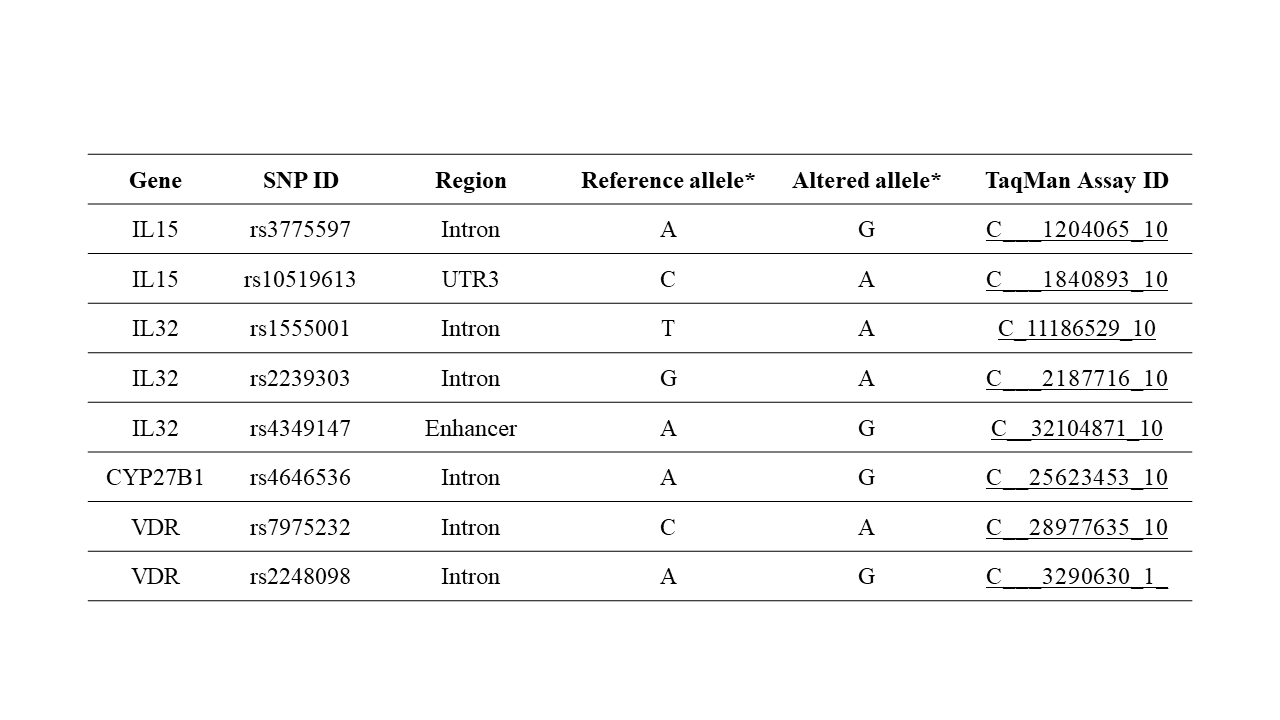
**

*Source: Reference SNP (rs) Report. National Library of Medicine. National Center for Biotechnology Information.

**Supplementary Table 5.** Genotype frequencies: Observed and expected for Patients, Healthy Controls and total study of populations/genes and the Hardy-Weinberg equilibrium test.

**
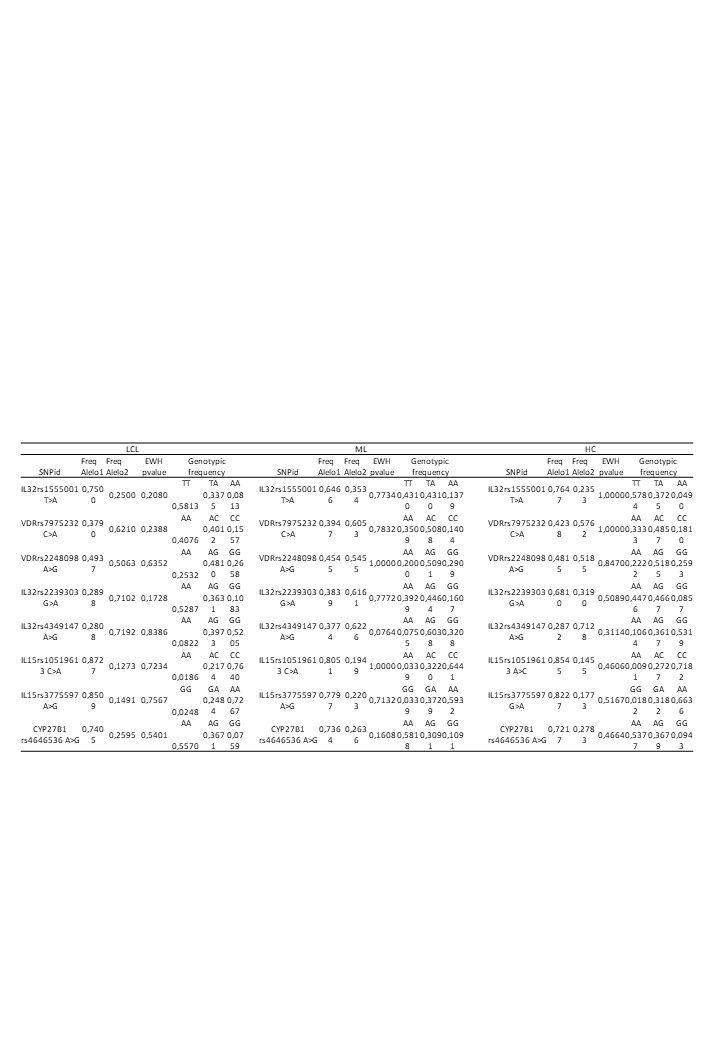
**

LCL= Localized Cutaneous Leishmaniasis

ML=Mucosal Leishmaniasis

HC= Healthy Controls

EWH= Hardy-Weinberg Equilibrium

**Supplementary Table 6.** Genotypes and alleles of single nucleotide polymorphisms in association with number of lesions in patients with cutaneous leishmaniasis


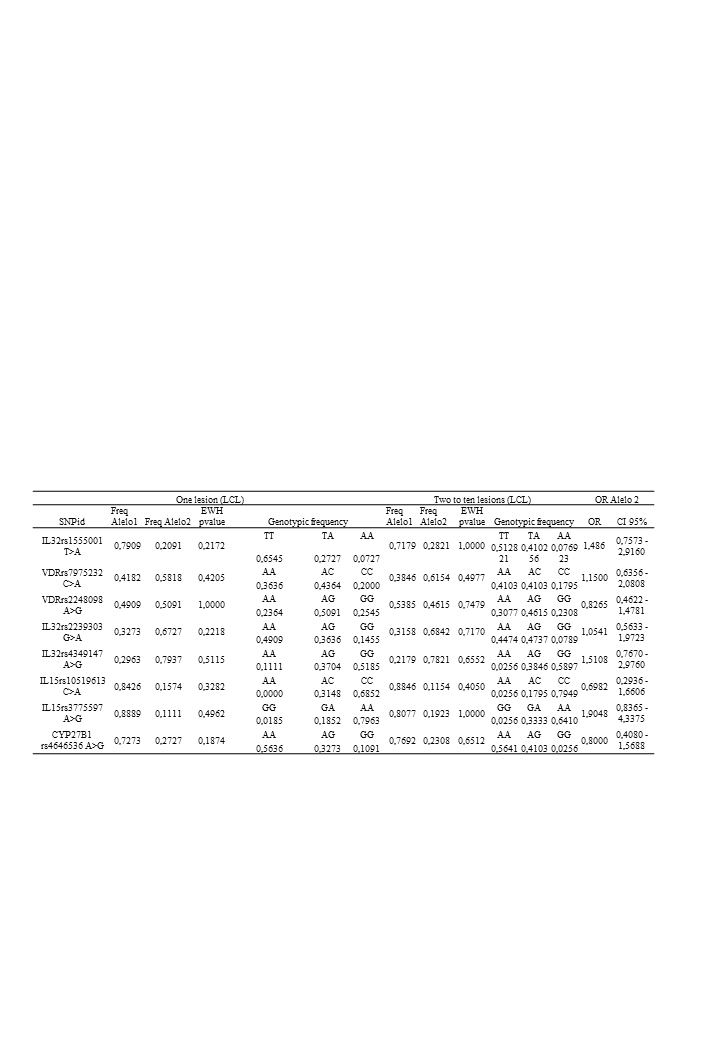


OR odds ratio, CI confidence interval; values with p≤0.05 considered as statistically signifcant and are represented by bold font.

**Supplementary Table 7.** Genotypes and alleles of single nucleotide polymorphisms in association with vitamin D levels in healthy controls and in patients with cutaneous or mucosal leishmaniasis


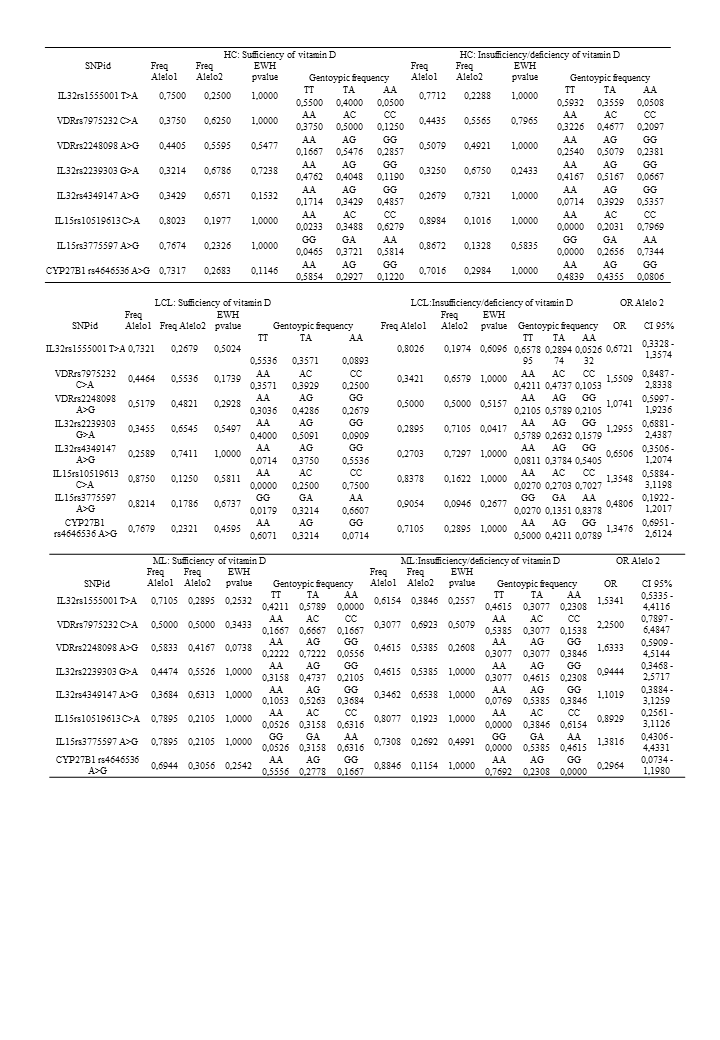


OR odds ratio, CI confidence interval; values with p≤0.05 considered as statistically signifcant and are represented by bold font.

LCL= Localized Cutaneous Leishmaniasis

ML=Mucosal Leishmaniasis

HC= Healthy Controls

**Supplementary Table 8.** Genotypes and alleles of single nucleotide polymorphisms in association with cure or failure in patients with cutaneous leishmaniasis.

**
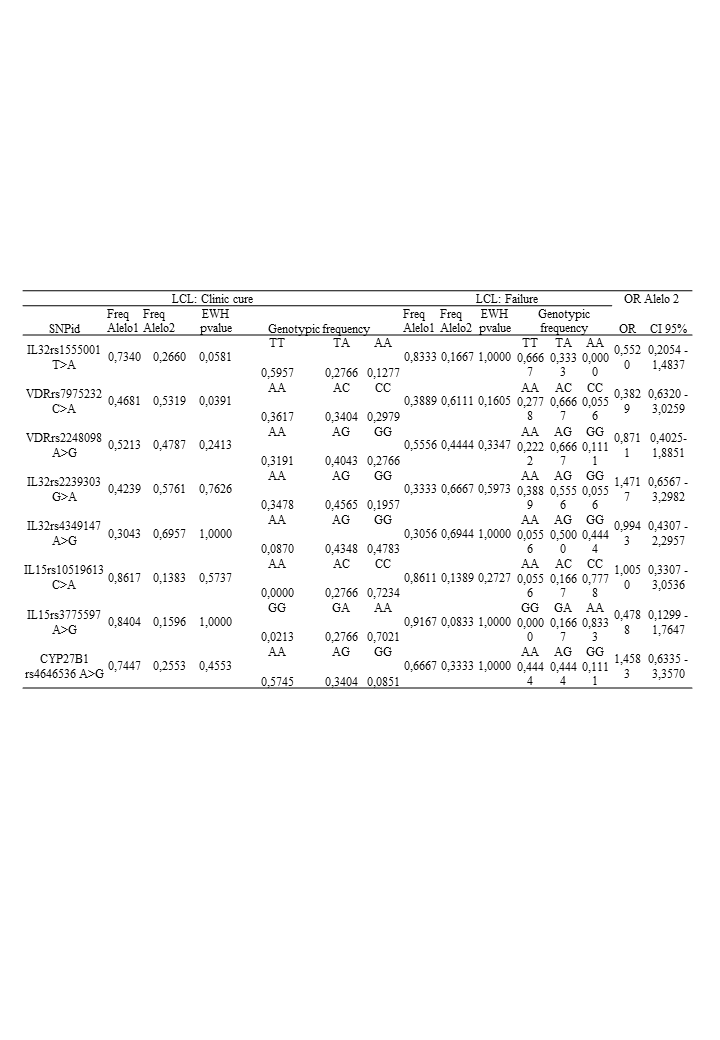
**

OR odds ratio, CI confidence interval; values with p≤0.05 considered as statistically signifcant and are represented by bold font.

LCL= Localized Cutaneous Leishmaniasis
